# The future of endemic and threatened birds of the Amazon in the face of global climate change

**DOI:** 10.1002/ece3.11097

**Published:** 2024-03-18

**Authors:** Kauê Felippe de Moraes, Marcela Guimarães Moreira Lima, Gabriela Silva Ribeiro Gonçalves, Pablo Vieira Cerqueira, Marcos Pérsio Dantas Santos

**Affiliations:** ^1^ Conservation Biogeography and Macroecology Laboratory – BIOMACRO Federal University of Pará Belém Brazil; ^2^ Graduate Program in Zoology Federal University of Pará Belém Brazil

**Keywords:** Amazon, birds, gap analysis, protected areas, species distribution models

## Abstract

The anthropogenic impacts on the environment, including deforestation and the escalating emissions of greenhouse gases, have significantly contributed to global climate change that can lead to alterations in ecosystems. In this context, protected areas (PAs) are pillars for biodiversity conservation by being able, for example, to maintain the viability of populations of endangered species. On the other hand, the species range shifts do not follow the limits of PAs, jeopardizing the conservation of these species. Furthermore, the effectiveness of PAs is consistently undermined by impacts stemming from land use, hunting activities, and illegal exploitation, both within the designated areas and in their adjacent zones. The objectives of this study are to quantify the impacts of climate change on the distribution of threatened and endemic birds of the Amazon biome, evaluate the effectiveness of PAs in protecting the richness of threatened birds, and analyze the representativeness of species within PAs. We found with our results that climate suitability loss is above 80 for 65% of taxa in the optimistic scenario and above 93% in the pessimistic scenario. The results show that PAs are not effective in protecting the richness of Amazonian birds, just as they are ineffective in protecting most of the taxa studied when analyzed individually Although some taxa are presented as “Protected,” in future scenarios these taxa may suffer major shrinkages in their distributions and consequently present population unviability. The loss of climatically suitable areas and the effectiveness of PAs can directly influence the loss of ecosystem services, fundamental to maintaining the balance of biodiversity. Therefore, our study paves the way for conservation actions aimed at these taxa so that they can mitigate current and future extinctions due to climate change.

## Graphical abstract

The manuscript addresses how climate change may affect the distribution of threatened bird species in the present and future. In addition, we seek to assess whether existing protected areas in the Brazilian Amazon are efficient in conserving climatically suitable areas in present and future scenarios.

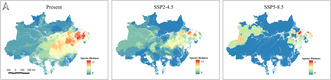

## 1 INTRODUCTION

Human‐induced alterations in climate patterns have resulted in shifts in temperature and precipitation patterns, contributing to the rise in extreme weather events worldwide (Anthony et al., 2012; Polade et al., 2014; Schneider & Held, 2001). Such events have direct impacts on global biota, as they affect aspects of phenology (Forrest, 2014; Lany et al., 2016), species behavior (Beever et al., 2017; McCann et al., 2017), morphology (Jirinec et al., 2021), and most importantly, cause serious impacts on species distribution, and therefore in the ecological interactions, altering ecosystem functioning (Bellard et al., 2012). The primary factors behind changes in global climate patterns are largely attributed to rapid socioeconomic development and the transformation of natural areas by human activities. These changes have outpaced the ability of species to adapt or migrate (Loarie et al., 2009; Román‐Palacios & Wiens, 2020).

Among the environments that will be most impacted by these changes in climate regimes are tropical forests (Gullison et al., 2007; Lewis, 2006; Mitchard, 2018; Wright, 2010; Zhang et al., 2021). Such environments demand a very stable overall water regime to maintain themselves and the species that inhabit those areas live close to their climatic limits (Khaliq et al., 2014). However, in the absence of coordinated conservation policies at both national and international levels, tropical forests may become sources of carbon in the atmosphere (Mitchard, 2018), moreover, increases in average temperature and changes in the pattern of precipitation regimes are expected (Gullison et al., 2007; Malhi et al., 2008; Scholze et al., 2006; Zhang et al., 2021). In this sense, climate change can lead to losses of ecosystem services primordial to the maintenance of tropical forests, such as seed dispersal, the germination success of these seeds, and, as consequence, the process of forest regeneration (Miranda et al., 2019; Wright, 2010). Furthermore, the declines in climatically suitable areas for species occurrence caused by climate change exert a direct influence on population dynamics(Chacón‐Prieto et al., 2021; Prieto‐Torres et al., 2021; Ribeiro et al., 2016). Individuals establish themselves in habitats where local environmental conditions are favorable to their survival and reproduction, and in response to changes in climate, species have altered their distribution patterns over generations (Rushing et al., 2020; Sutton et al., 2022). These changes in distribution patterns caused by climate change can lead to massive population declines and even species extinction (Román‐Palacios & Wiens, 2020; Şekercioğlu et al., 2012; Urban, 2015).

In the face of adverse and extensive environmental impacts, it is imperative for species to possess climatically stable habitats, facilitating their adaptation and the preservation of equilibrium with climatic conditions. This, in turn, mitigates the risks of extinction attributable to climate change (Jetz et al., 2007; Suggitt et al., 2018). In this context, protected areas (PAs) are one of the pillars of biodiversity conservation (Watson et al., 2014), for being able to maintain the viability of populations of threatened species or potentially impacted by human occupation (Fagundes et al., 2016; Verissimo et al., 2011). Because of this, over the years more and more threatened species have their distributions increasingly represented in PAs, as a result of the increased implementation of these types of areas (Pacifici et al., 2020; Venter et al., 2014). PAs may be the only way to ensure favorable microclimatic conditions for species in anthropized or heterogeneous regions (Mackey et al., 2012; Nepstad et al., 2008). Moreover, protecting the species' habitat, even if they are exposed to climate change, helps to increase their resilience to this impact (Ribeiro et al., 2016).

On the other hand, the persistence of species within PAs may be compromised, since PAs are fixed over time and space and do not follow species range shifts (Hannah et al., 2013; Lemes & Loyola, 2013). Additionally, the efficacy of PAs is consistently jeopardized by internal challenges, including land use, illicit hunting of species, financial constraints, and management‐related issues, thereby posing a threat to biodiversity conservation. (Jones et al., 2018; Kauano et al., 2017; Rija et al., 2020). Thus, assessing the representativeness of species' ranges within PAs is increasingly important for policy and funding discussions targeting biodiversity conservation (Coad et al., 2019; Pacifici et al., 2020).

Birds are recognized as an excellent biological group to assess the integrity of systems due to their diversity, both by encompassing species tolerant to anthropic impacts, as well as sensitive species, with specialized niches (Şekercioğlu et al., 2012; Temple, 1989). In this sense, they perform several significant ecological services within an ecosystem, including seed dispersal, pest control, and pollination (Nathan & Muller‐landau, 2000; Pizo & Galetti, 2000), which differ according to geographic location, occupying different niches, thus being sensitive to changes in the environment (Jetz et al., 2007; Sekercioglu, 2006). Knowing and understanding the geographic and ecological distribution of birds has been one of the tools of conservation biology for the implementation of protection strategies for endangered species (Godown & Peterson, 2000).

In order to quantify the impacts of climate change on the distribution of threatened and endemic birds of the Brazilian Amazon, the methodology of species distribution modeling is seen as a suitable alternative, because from the occurrences of species and measures of climate variables, it is possible to trace areas of climatic suitability for each taxa in present and future scenarios (Andrade et al., 2020; Elith & Leathwick, 2009; Guisan & Zimmermann, 2000; Peterson, 2006; Peterson et al., 2011). Furthermore, our study seeks to evaluate the effectiveness of PAs in protecting the taxonomic richness of threatened birds through null models (Lemes et al., 2013; Ribeiro et al., 2016) and, finally, to analyze the representativeness of individual taxa within the PAs, from taxa distributions in the baseline and two future climate scenarios, using gap analysis (Rodrigues et al., 2004).

## 2 METHODOLOGY

### 2.1 Study area

**FIGURE 1:**
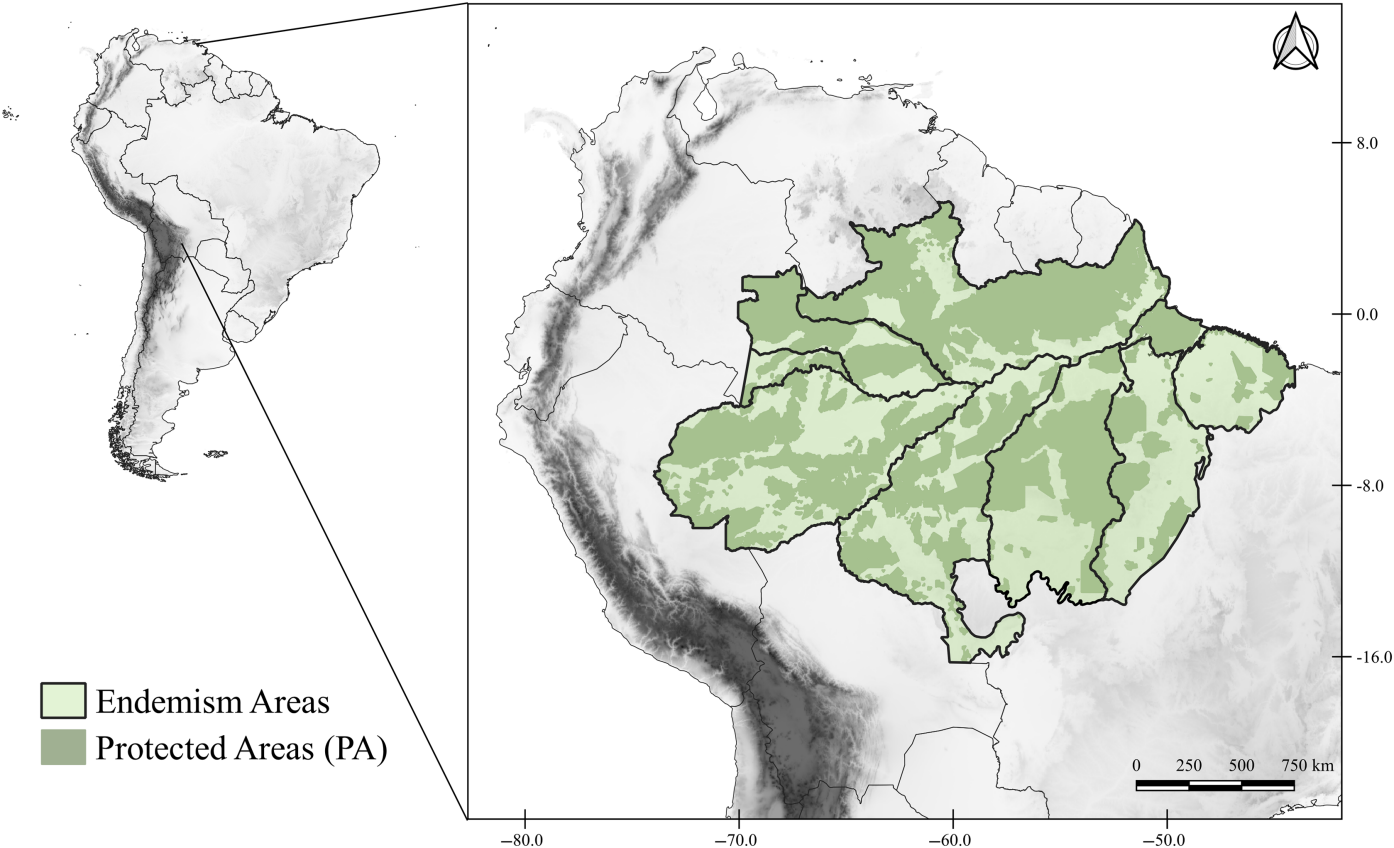
Map of the study area showing the location of the Amazon. In light green with black lines, the Brazilian Amazon map, is divided into eight Centers of Endemism, plus the Marajó Island. In dark green, the existing protected areas in the Amazon. In white, the continent that shelters the Biome. Broadview of the location of the Amazon in South America. Shapefile provided by the World Database on Protected Areas (WDPA), the Amazon Network of Geo‐referenced Socio‐environmental Information, and the Ministry of the Environment (MMA).

The study area corresponds to the Brazilian Amazon, which covers about 4,196,943 km^2^ of the Brazilian territory (MMA, 2021). The Brazilian Amazon has an average rainfall index of 2300 mm/year, which can reach 5000 mm/year in the western portion of the biome (Marengo & Nobre, 2009). The annual mean temperature varies significantly across its different areas due to the vast expanse of the biome. In regions near the equator line, such as the North, the annual mean temperature is higher, often exceeding 26°C. However, in the southwestern Amazon, the annual mean temperatures tend to be slightly milder, averaging around 24°C (Alvares et al., 2013; Nobre et al., 2009). In this region, there is a predominance of yellow latosols and ultisols (Vieira & Santos, 1987), with a pattern of weathering, and physical characteristics suitable for agricultural use. However, this type of soil presents strong nutritional limitations (Vieira & Santos, 1987) (Figure 1).

### 2.2 Target taxa and occurrence data obtaining

We selected 47 Amazonian endemic taxa that have been assessed under some threatening status, according to the Red Book of Threatened Species in Brazil (ICMBio, 2018), in addition to two newly described species (*Megascops ater* and *Megascops stangie*), which have restricted distributions (Dantas et al., 2021) and preliminary analyses suggest that they may be included in upcoming official lists of threatened species, as they fulfill several criteria outlined in the IUCN assessment forms, thus totaling 49 taxa (Table S1). The database was produced from the compilation of georeferenced occurrence points obtained through the following platforms: Global Biodiversity Information Facility (GBIF, Table S2), eBird (https://ebird.org/home), Xeno‐Canto (https://xeno‐canto.org/), Vertnet (http://www.vertnet.org/index.html), SpeciesLinks (https://specieslink.net/), and the Biodiversity Portal—ICMBio (https://portaldabiodiversidade.icmbio.gov.br/portal/). In addition, we used occurrence information obtained from the ornithology collection of the Museu Paraense Emilio Goeldi (MPEG). To mitigate uncertainties or sampling errors, the database underwent an evaluation by experts in the distribution of Amazonian bird taxa (see Appendix S1 for taxa nomenclature and cleaning the taxa occurrence records).

### 2.3 Environmental variables

We used 19 bioclimatic variables for the baseline (1970–2000; hereafter known as the “present” scenario) and future (2081–2100) scenarios at the spatial resolution of 2.5 arcminutes (~5 km), obtained from the WorldClim 2.0 platform (Fick & Hijmans, 2017). To assess the effect of climate change on threatened Amazonian birds in the future, we used two alternative shared socioeconomic pathways (SSPs) scenarios from CMIP6 climate projections (Eyring et al., 2016), where SSP2‐4.5 is presented as an optimistic scenario of CO^2^ emissions and SSP5‐8.5 being a pessimistic scenario (See Appendix S2 for SSPs nature and details). For this study, we selected five global climate models (GCMs) after a cluster analysis, as proposed by Varela et al. (2015), aiming to maximize the uncertainties among climate models. The selected GCMs were BCC‐CSM2‐MR, CanESM5, IPSL‐CM6A‐LR, MIROC6, and MIROC‐ES2L. Aiming to mitigate the redundancy of the climate variables, we performed a principal component analysis (PCA), where the first six axes that explain 95% of the original variation of the climate variables were selected as predictors of the response functions (de Marco & Nobrega, 2018). In order to maintain the dimensionality of the climate data throughout the temporal scenarios, the coefficients obtained from the PCA were used to predict the scores of the future climate data (Table S3) (Sillero & Barbosa, 2021).

### 2.4 Modeling and model evaluation

In this study, we used five algorithms to generate species distribution models (SDMs): Maxent (MXS), Bayesian Gaussian Process (GAU), Random Forest (RDF) Support Vector Machine (SVM), and Generalized linear Models (GLM) (Breiman, 2001; Golding & Purse, 2016; Guisan et al., 2002; Phillps & Dudík, 2008; Tax & Duin, 2004) (see Appendix S3 for algorithm details and pseudo‐absence production). The performance of the models was evaluated from two data partitioning approaches. For taxa with 15 or more unique occurrences, we used a geographically structured non‐random cross‐validation method called checkerboard (Roberts et al., 2017), which divides the data into two subsets: while one subset is fitted, the other is used for evaluation of the first subset and vice versa, alternating the subsets for fitting and evaluation (Andrade et al., 2020). In pursuit of the optimal model fit, we selected the calibration area for each taxon under study based on the endemism centers where true occurrences are present. Despite the interfluves not constituting complete barriers for some taxa, the endemism centers directly influence the composition of birds throughout the biome, governing the distribution pattern of endemic taxa in the Amazon. For taxa with unique occurrences below 15 points, we used the k‐fold cross‐validation methodology (Roberts et al., 2017; Wiens et al., 2008) (See Appendix S4 for checkerboard and K‐fold validation approaches). Among the studied taxa, eight had the data partitioned using the K‐fold method, and 41 taxa with checkerboard. After the data partitioning process, the models were evaluated using two metrics: (1) the AUC (area under curve) (Fielding & Bell, 1997), a non‐threshold dependent evaluation measure that uses the average of true positives (sensitivity) among the possible false positive values (specificity), generating a measure of fit of the models produced; (2) Jaccard's Similarity Index (Jaccard, 1908), which calculates the similarity between predictions and observations from partitioned data. The Jaccard Similarity Index ranges between 0 and 1. The closer to 1 the index shows, the better the correspondences between observations and predictions (Leroy et al., 2018) and, consequently, the better evaluated the models will be.

Finally, we generated the consensus models through the Ensemble Forecasting approach (Araújo & New, 2007; Lima‐Ribeiro & Diniz‐Filho, 2012). In this approach, the models are generated from the simple average of the algorithms that performed above the general average of all analyzed algorithms. This procedure was made for both present and future climate scenarios (SSP2‐4.5 and SSP5‐8.5). All procedures to generate and evaluate the models were performed using the ENMTML package (Andrade et al., 2020) using R software v 4.0.2 (R Core Team, 2021).

After initial analysis of the species distribution models and the evaluation of Amazonian taxa experts, six taxa were excluded from the analyses for presenting distribution patterns inconsistent with the reality of their respective groups in relation to their niches and environmental preferences. The taxa removed were: *Campylorhamphus cardosoi*, *Neomorphus squamiger*, *Phaethornis aethopygus*, *Pyrrhura anerythra*, *Stigmatura napensis napensis*, and *Serpophaga hypoleuca pallida*. In addition, the taxa *Myrmotherula klagesi* and *Picumnus varzeae* had their future distribution models limited by the climatically suitable areas of the present models, because they are restricted to floodplain areas and occur only near riparian areas (Cohn‐Haft et al., 2007). Thus, 43 of the 49 taxa selected for this study were analyzed.

### 2.5 Overlapping species distribution models and land use models

Because the studied taxa are directly dependent on specific vegetation areas and aiming to reduce the extrapolation of climate models, we chose to overlay the taxa distribution available at 1 km spatial resolution with projections from 2015 to 2100 and is divided into two subsets: The land use and land cover (LULC) dataset, and the global land dataset based on plant functional types (PFT), containing 20 land use types (Chen et al., 2022). For this investigation, we modified the spatial resolution to 2.5 arcminutes to align the LULC data with the resolution of the Environmental variables. Within the forest cover types available in the models, we selected the categories that influence the distribution of the target species, these being: Broadleaf evergreen tree, tropical; Broadleaf deciduous tree, tropical; Broadleaf deciduous shrub, temperate (see Appendix S5 for further information about the use of LULC models).

To obtain the taxa richness maps in the different scenarios, we performed stacked species distribution models (S‐SDM). For all overlays, we use the QuantumGis 2.8 “raster calculator” tool.

### 2.6 Spatial dynamics and effectiveness of protected areas

To perform the analyses of spatial dynamics and effectiveness of PAs, the continuous models were transformed into binary ones, starting with the use of a threshold based on Jaccard's similarity index. Thresholds are chosen based on the fitness value that maximizes a given metric (Andrade et al., 2020). For this study, we selected a threshold that uses a fitness value that provides the highest Jaccard value. After the binarization process of the models, we evaluated the percentage of loss or gain of climatically suitable area for each taxa, calculating the area for each scenario (present and future) and then calculating the difference between the areas in the present and future.

To represent the PAs in the Amazon biome, we considered three PA categories, which are the following: strictly protected areas (SPAs), sustainable use areas (SUAs), and indigenous territories (ITs). These data were obtained through the compilation between the World Database on Protected Areas (WDPA) (https://www.protectedplanet.net/, UNEP‐WCMC), the Amazon Network of Geo‐referenced Socio‐environmental Information (https://www.raisg.org/) and the Ministry of Environment (http://mapas.mma.gov.br/i3geo/datadownload.htm). Thus, we selected a final set of 347 PAs (see Appendix S6 for further information about PAs in Brazil and the selection of PAs for the study).

To assess the PAs effectiveness in protecting taxa richness, we used a null model approach, which assesses the ability of PAs to retain greater taxa richness than would be expected by chance (Ribeiro et al., 2016; Velazco et al., 2020). In this approach, PAs were randomly relocated 999 times within our study area, maintaining their size, shape, and orientation (Ribeiro et al., 2016). At each randomization, we calculated the average richness value based on the cells encompassed by each PA for all scenarios (current and future). PAs are considered effective for taxa protection when the taxa richness within a given PA is greater than the taxa richness of that randomly allocated PA in at least 95% of randomizations (*p* < .05) (Ribeiro et al., 2016). Data were analyzed using the raster (Hijmans & Van Etten, 2012) and dplyr (Wickham et al., 2023) packages using R software v 4.0.2.

To evaluate how the PAs in the Amazon have performed in the conservation of taxa, we used a gap analysis (Rodrigues et al., 2004). This analysis proposes to classify taxa from conservation targets based on the representativeness of the taxa distributions in PAs. These targets propose that species with <1000 km^2^ of distribution should be 100% within PAs, while species with >250,000 km^2^ should be at least 10% within PAs. Values between these two gradients are calculated from an interpolation using a logarithmic transformation (Rodrigues et al., 2004).

The PAs were divided into three scenarios for the analysis: the SPA scenario, using only SPAs; the SPA + SUA scenario, using SPAs and SUAs; the SPA + SUA + IT scenario, using SPAs, SUAs and IT areas (Fagundes et al., 2016). As for the protection goal, species were classified as (i) protected when reaching 90% of the goal; (ii) partially protected when reaching >20% to <90% of the goal; (iii) gap when reaching up to 20% of the goal; and (iv) not protected when the percentage is 0 (Frederico et al., 2018; Velazco et al., 2022). The analysis was used to assess the representativeness of PAs on the time scales of the present scenario and the two future scenarios. The data were analyzed using R software v 4.0.2.

## 3 RESULTS

After database cleaning procedures, SDMs were built from 2767 unique occurrences with *Dendrexetastes paraensis paraensis* being the taxon with the fewest occurrence points (8) and *Tinamus tao tao* the taxon with the most unique occurrence points (518). The average Jaccard similarity index for the taxa where K‐fold was used was 0.95 (ranging from 0.87 to 1), while the average AUC value was 0.95 (ranging from 0.93 to 0.98) (Table S1). For taxa with data partitioned using the checkerboard method, the mean Jaccard similarity index presented was 0.85 (ranging from 0.70 to 0.87), while the mean AUC value was 0.94 (ranging from 0.76 to 0.99) (Table S1).

The species distribution models show that in the optimistic scenario, 29 of the studied taxa may present a loss of climatic suitability higher than 80% for the whole Brazilian Amazon, with 20 of them having no climatically suitable areas in the future (area loss higher than 99%). On the other hand, in the pessimistic scenario, 40 taxa have a loss of climatic suitability >80%, with 36 taxa presenting no climatically suitable areas (Figure S1; Table S4).

Of the taxa lacking suitable areas in any of the future scenarios (optimistic and pessimistic), four of them (*Celeus torquatus pieteroyensi*, *Dendrexetastes paraensis paraensis*, *Piculus paraensis*, and *Picumnus varzeae*) have threatening status “Endangered (EN)” and one (*Crax fasciolata pinima*) was assessed as “Critically Endangered,” according to the Red Book of Threatened Species (ICMBio, 2018). In addition, the taxon *Psophia obscura*, threatening status “critically endangered” (CR), presents considerable loss of suitability in the optimistic scenario (78%), and if the projections come true, it may be at risk of extinction in a pessimistic climate scenario, by presenting loss of 99% of climatically suitable area.

**FIGURE 2:**
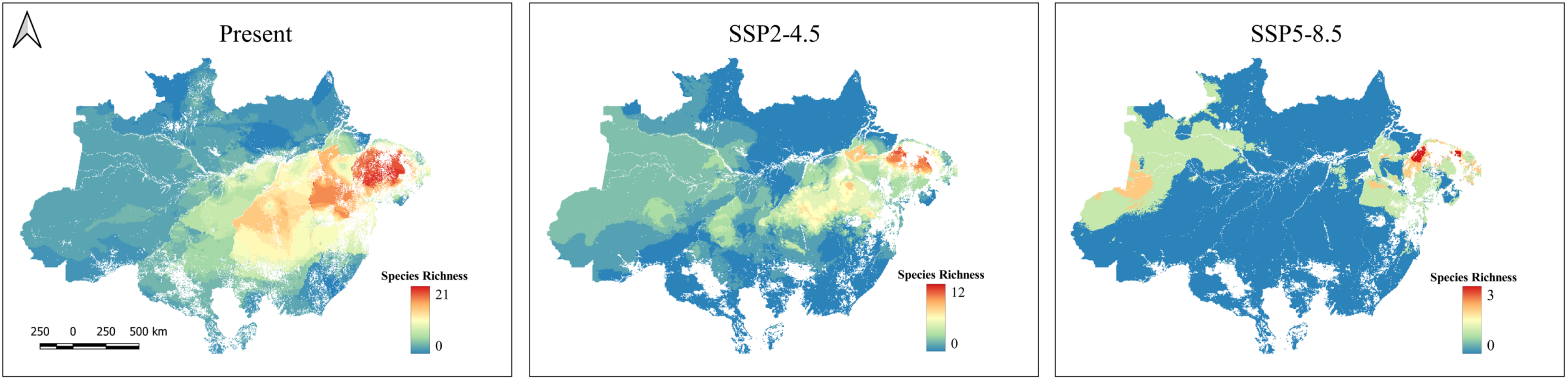
Map of threatened bird taxa richness in the Brazilian Amazon for both present and future (SSP2‐4.5 and SSP5‐8.5). In red are the areas of greatest richness regardless of the climate scenario. In dark blue, the projected areas lacking climatic suitability for the taxa addressed in the study. Shapefile of the domain provided by MMA (Ministério do Meio Ambiente do Brasil‐Ministry of the Environment).

According to the models for the present scenario, the regions with the highest taxonomic richness of threatened taxa are located in a small part of the eastern Amazon region. In the future scenarios, we see a decrease in richness with loss of 9 taxa in the maximum overlap range in SSP2‐4.5 and 17 taxa in SSP5‐8.5. We verified a progressive decrease in richness from the present to future scenarios, mainly concentrated in Belém and Xingu Centers of Endemism (Figure 2).

**FIGURE 3:**
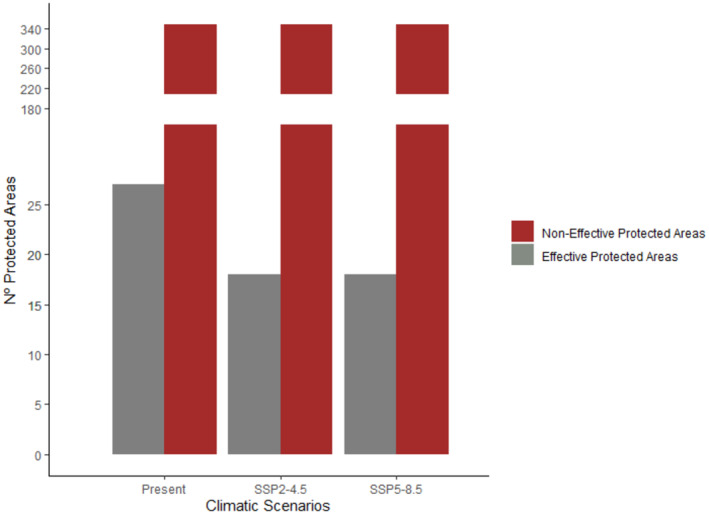
Number of effective (gray) and non‐effective (red) protected areas for the conservation of threatened bird richness in the Amazon under the present, optimistic future (SSP2‐4.5) and pessimistic future (SSP5‐8.5) climate scenarios, according to the null model analyses.

By analyzing the effectiveness of PAs through null models, our results show that, in the current scenario, out of the 347 PAs existing in the Brazilian Amazon, only 7.7% (27) are effective in conserving the richness of endemic birds threatened with extinction. In both the optimistic and pessimistic future scenarios, the percentage of effective PAs remains constant at 5.2%, encompassing 18 PAs capable of safeguarding our target taxa (Figure 3).

**FIGURE 4:**
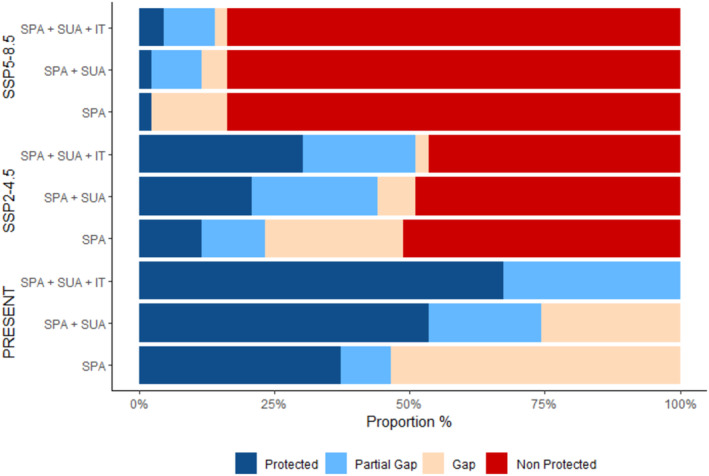
Proportion of taxa within protection categories from the three climate scenarios: strictly protected areas (SPAs), sustainable use areas (SUAs), and indigenous territories (ITs), according to the gap analysis. In dark blue, the proportion of taxa considered Protected; in light blue, the proportion of taxa considered partial gap; pink color represents the “gap” category; and red represents the proportion of taxa considered “not protected” and taxa lacking climate suitability in the respective climate scenarios.

From the gap analysis, we can see that the integral protection areas (SPA) alone are not sufficient to protect the taxa in isolation. In this category of protection (SPA), over 50% of the taxa are classified as gaps regardless of the climate scenario (Figure 4). By adding the sustainable use areas (SPA + SUA), one can see an increase in the number of taxa classified as protected in both current scenario and SSP2‐4.5 (Table 1). In the pessimistic future scenario, the number of protected taxa remains stable (Table 1).

**TABLE 1:** Number of taxa within protection categories from the three climate scenarios: strictly protected areas (SPAs), sustainable use areas (SUAs), and indigenous territories (ITs), according to the gap analysis. Taxa lacking climate suitability in the future scenarios were considered as unprotected in the gap analysis.

Category	Present			SSP2‐4.5			SSP5‐8.5		
	SPA	SPA + SUA	SPA + SUA + IT	SPA	SPA + SUA	SPA + SUA + IT	SPA	SPA + SUA	SPA + SUA + IT
Protected	16	23	29	5	9	13	1	1	2
Partial gap	4	9	14	5	10	9	0	4	4
Gap	23	11	0	11	3	1	6	2	1
Not protected	0	0	0	22	21	20	36	36	36
Total	43	43	43	43	43	43	43	43	43

Considering all PA categories (SPA + SUA + IT) and taking into account only taxa presenting climatic suitability for the future, it can be seen that taxa have their distributions well represented within PAs, with 67% of taxa protected in the present scenario and 56% protected in the optimistic future (Table 1). On the other hand, in a pessimistic scenario, even considering all PA categories (SPA + SUA + IT), only two taxa would be considered protected. Some taxa deserve to be highlighted, such as *Megascops ater* and *Phlegopsis nigromaculata paraensis*, not presented as “protected” in any of the PA categories and any climate scenario (Table 1, Table S5).

## 4 DISCUSSION

Our results suggest a worrying scenario for the conservation of Amazonian birds. On the feasible effects of climate change on threatened and endemic birds of the Brazilian Amazon, both in current and in two alternative future scenarios (pessimistic and optimistic), we observed a taxa loss of 46% in the optimistic scenario, while in the pessimistic scenario, 83% of taxa may face extinction by the year 2100. This general scenario becomes worse since our results also indicate that the PAs currently existing in the Amazon biome are not very effective in protecting the richness of endemic birds. On the other hand, by analyzing the taxa representation within the individual PAs, we realize the importance of combining different categories of PAs and indigenous lands to ensure favorable climatic and environmental conditions for taxa conservation.

Some studies have already predicted an increase in the speed of changes in temperature and precipitation for the tropical region, which may lead to extreme drought and warming conditions, as well as large‐scale “savannization” events, mainly in southern and eastern Amazon (Garcia et al., 2014; Loarie et al., 2009; Nobre et al., 2016). Thus, the intensity and speed of these changes will challenge the ability of taxa to adapt to new climatic conditions and disperse to suitable areas (Parmesan, 2006). In addition to occurring in areas where the greatest climate changes are expected, many of the taxa studied are geographically restricted to the interfluves of the large Amazonian rivers and thus are expected to be even more sensitive to climate change (Bozinovic et al., 2011; Foden et al., 2013; Pacifici et al., 2015; Sheth & Angert, 2014), by having reduced population sizes or lower genetic diversity (Collevatti et al., 2013; Morueta‐Holme et al., 2010; Pauls et al., 2013). Species having distribution restrictions are also vulnerable to non‐climatic stressors, such as the Allee effect and population stochasticity, that is, even small variations in climate can expose all populations to physiological stress (Oswald et al., 2011; Sexton et al., 2009).

In addition, our results corroborate a pattern that is becoming solid, which is the environmental impacts leading to abrupt climate changes around the world, and consequently directly influencing habitat loss and future distributions of species. This is a phenomenon already recorded both in the Amazon and other Neotropical regions (Escobar‐Luján et al., 2022; Prieto‐Torres et al., 2021), in addition to the United States (Northrup et al., 2019), Europe (Virkkala, 2016), and Oceania (Porfiro et al., 2016). In fact, if the projections come true, de Moraes et al. (2020) also documented a considerable loss of climatically suitable areas, potentially resulting in the disappearance of endemic bird taxa in the eastern Brazilian Amazon by the year 2050. This shows that the effects of climate change are extremely worrying, with serious effects on the extinction of species, requiring immediate action in terms of public policies for the Amazonian biota conservation. The urgency for conservation‐oriented actions is further underscored when we recognize that the habitat loss for bird taxa, induced by climate change, is an impact not confined solely to the Amazon but is also prevalent in Cerrado (Borges & Loyola, 2020), Atlantic Forest (Loiselle et al., 2010), and Caatinga biomes (Gonçalves et al., 2023). Furthermore, it can also be observed in other organisms such as mammals (Baisero et al., 2020), lizards (Teixeira et al., 2022), and invertebrates (Giannini et al., 2012).

The loss of climatically suitable areas in the face of climate change can also directly influence the loss of ecosystem services, which are vital to maintaining the balance of natural and urban ecosystems, both at regional and global scales (Mooney et al., 2009; Weiskopf et al., 2020). In this context, several bird species fulfill an important role by dispersing seeds through their frugivorous habits (Caves et al., 2013). According to our results, frugivorous taxa such as *Aratinga solstitialis*, *Guaruba guarouba*, and *Pteroglossus bitorquatus bitorquatus* may lose up to 80% of their climatically suitable areas under an optimistic scenario, while the taxa *Crax fasciolata pinima* and *Tangara velia signata* may not find suitable areas in the evaluated future scenarios (Table S4). Impacts on the composition of frugivorous species of an ecosystem can drastically affect the diversity of forests, as well as their regeneration processes, and also plant cover structuring for decades, since they are able to disperse a large amount and diversity of seeds (Mota et al., 2022). Studies such as that of Miranda et al. (2019), present similar results regarding the total loss of suitable areas of frugivorous species and further conclude that seed disperser species may be more sensitive to climate change. If our projections come true, climate change will directly impact the composition of species fundamental to seed dispersal in the Amazon biome and may negatively influence vegetation cover patterns in the region.

Among the taxa assessed as critically endangered and with substantial loss of suitable area, we have *Crax fasciolata pinima*, a forest taxa that inhabits the most critical part of the Amazon: the Belém endemism center, which comprises the states of Maranhão and Pará (Alteff et al., 2019). In this area, were detected the highest levels of habitat loss in the entire Amazon basin. For instance, only in 2021, the Pará state had the largest deforested area in Brazil (402,4992 ha) corresponding to 24% of the total deforestation in this year, whereas Maranhão state ranked fourth in the same rank (MapBiomas, 2022). The bird *Crax fasciolata pinima* is considered rare and one of the least known taxa with a total population estimated at <100 individuals, thus being on the verge of extinction (Lees et al., 2013). Furthermore, this taxa went 40 years with no records, being rediscovered only recently, in four localities (Alteff et al., 2019). One of these localities is the Gurupi Biological Reserve, which, despite being a PA, has been suffering from illegal logging, with 180.9 ha deforested only in 2021 (Alteff et al., 2019; MapBiomas, 2022). The importance of this area has been highlighted previously by de Moraes et al. (2020), and here we reinforce that, of the 39 studied taxa, 19 are found in this PA, including *Psophia obscura*, a species also assessed as Critically Endangered.

Another worrying finding in our results is the ineffectiveness of PAs in protecting taxa richness. Currently, more than 42% of the Brazilian Amazon is covered by PAs (Verissimo et al., 2011), 12% more than the goals proposed by the National Biodiversity Council (CONABIO, 2013) for the year 2020. Although these values are optimistic for taxa conservation in general, our null model results show that the existing PAs in the Amazon biome may not be effective for the richness conservation of threatened Amazon birds, both in current and future scenarios. This discrepancy may be attributed to the fact that PAs are built based on political issues, being implemented in areas with lower maintenance costs or low economic interest, without effectively prioritizing the responsibility of conserving biodiversity (Venter et al., 2014). Additionally, the implementation of PAs in the Amazon is relatively recent, and because of this, there are few studies supporting the management and creation of other PAs (Oliveira et al., 2017).

An important factor that weakens the effectiveness of PAs is the lack of these areas in the eastern portion of the Amazon. This is an area with high rates of deforestation and the highest number of endangered taxa, in addition to the lack of connectivity between fragments. Our results corroborate the fact that the eastern region has the greatest richness of threatened bird taxa and demonstrate a progressive loss of this richness starting with future climate scenarios. Despite having fewer PAs than the western region, the SPA, and SUA adjacent to indigenous lands (such as the Gurupi mosaic, for instance) in the eastern portion of the Amazon play a crucial role in supporting the occurrence of these threatened species. These areas are particularly significant for the conservation of endemic and endangered avian species in the eastern sector of the Amazon biome (de Moraes et al., 2020).

These challenges are not limited solely to Brazilian PAs, as similar deficiencies are prevalent in the majority of PAs within the Neotropical region (Baldi et al., 2017; Collen et al., 2008; Saura et al., 2017). The ineffectiveness of PAs in protecting species richness was also recorded for plants in biomes like Cerrado (Velazco et al., 2019), and Caatinga, where cactus species were unprotected by the PA network (Carvalho et al., 2022). Prieto‐Torres et al. (2018), assessing PAs in several Neotropical dry forests, found that the current network covers <15% of the ranges of 80% of bird taxa. PAs in the Amazon were also not effective for mammals, where only 28% of the studied PAs were considered effective in protecting species richness (Ribeiro et al., 2016).

Integral Protection Areas are ineffective in protecting taxa, when analyzed independently. Such gaps are noticeable in the three climatic scenarios and demonstrate that most of the studied taxa are poorly represented in this type of PA. However, these results do not mean that the PAs are totally ineffective for taxa conservation. When uniting areas of conservation units with indigenous lands, most of the taxa are classified in the “Protected” goal. These results corroborate with the study of Fagundes et al. (2016). In this study with turtles, the authors found that, when analyzing the protection gaps of PAs in the Brazilian Amazon, only the SPAs are ineffective to retain the distribution of taxa within their territories, and when adding the SUA and ITs most taxa were considered “protected”. The same results can be observed for other groups, such as fishes (Frederico et al., 2018).

Some species such as *Penelope pileata*, *Rhegmatorhina gymnops*, and *Synallaxis kollari* had area losses >90% and are still classified as “Protected” or “Partial Gap” according to the gap analysis. This phenomenon occurs due to the fact that much of the species' area losses occur in areas that have no protection whatsoever, increasing the proportion of suitable areas within PAs relative to total areas (Pacifici et al., 2020). This kind of result also supports the idea that, although some taxa suffer major shrinkages in their distributions, PAs still serve as refuge areas, as their suitable areas remain conserved within PAs (Berteaux et al., 2018; de Moraes et al., 2020). The results of the gap analysis also support the importance of indigenous lands for conservation, which have an essential role in protecting biodiversity and the effects of climate change (Oliveira et al., 2017; Soares‐Filho et al., 2010) by preventing anthropic activities from developing within these fragments. In the Amazon biome, indigenous lands play a key role in protecting species, especially in the eastern portion, sheltering endemic species in the midst of highly anthropized areas (de Moraes et al., 2020). Despite the importance of PAs, the current Brazilian government has anti‐preservation policies, and a legislative bill to reduce the protection status of indigenous lands and the release of mining activity (Villén‐Pérez et al., 2020). Such measures could further harm the conservation of our biodiversity.

In contrast, although species such as *Aratinga solstitialis*, *Chamaeza nobilis fulvipectus*, *Pyrrhura coerulescens*, and *Xiphocolaptes carajaensis* are classified as “protected” species in the current scenario, their projected climatically suitable areas are relatively restricted in at least one of the evaluated scenarios. Additionally, those areas present a historic population decline due to environmental impacts caused in the past (ICMBio, 2018). Although PAs play a role in the conservation of taxa living within their boundaries, biodiversity within these areas suffers direct and indirect pressures, such as the isolation of species due to high fragmentation of the surrounding unprotected landscape (Laurance et al., 2012). Furthermore, PAs are not immune to illegal activities such as vegetation suppression, illegal fishing, and hunting, among other activities that directly impact biodiversity (Kauano et al., 2017). In 2021 alone, the Brazilian Amazon was the most deforested biome, representing 59% of the total, also concentrating 73% of deforestation occurring within PAs (MapBiomas, 2022).

## 5 CONCLUSION

Our future projections for Amazon endemic birds present a feasible fate that is not restricted to this taxonomic group. The effects of climate change could lead to devastating biodiversity losses in all known ecosystems. In this context, PAs, which have a fundamental role in the conservation of taxa, have shown inefficiency in the conservation of taxa richness due to political biases in their creation and poor management. However, our results also call attention to the importance of indigenous lands and sustainable use conservation units in the conservation of taxa on an individual basis. The results provided here can be a first step towards a more comprehensive assessment of the vulnerability of Amazonian endemic taxa to climate change and can guide conservation decisions aimed at minimizing the negative effects of climate change on species.

## AUTHOR CONTRIBUTIONS

**Kauê Felippe de Moraes:** Conceptualization (equal); data curation (lead); formal analysis (equal); investigation (equal); methodology (equal); software (equal); validation (lead); visualization (equal); writing – original draft (lead); writing – review and editing (equal). **Marcela Guimarães Moreira Lima:** Conceptualization (equal); data curation (equal); investigation (equal); methodology (equal); supervision (equal); validation (equal); visualization (equal); writing – review and editing (equal). **Gabriela Silva Ribeiro Gonçalves:** Conceptualization (equal); data curation (equal); formal analysis (equal); investigation (equal); methodology (equal); software (equal); validation (equal); visualization (equal); writing – review and editing (equal). **Pablo Vieira Cerqueira:** Data curation (equal); methodology (equal); validation (supporting); writing – review and editing (equal). **Marcos Pérsio Dantas Santos:** Conceptualization (equal); funding acquisition (equal); project administration (equal); resources (equal); supervision (equal); writing – review and editing (equal).

## FUNDING INFORMATION

We declare that this study was partly financed by the Coordenação de Aperfeiçoamento de Pessoal de Nível Superior—CAPES (00.889.834/0001‐08) that provided financial support granted to KFM (process #88887.494045/2020‐00) as a scholarship, and the Conselho Nacional de Desenvolvimento Científico e Tecnológico (CNPq) that provides financial support granted to MPDS with research productivity fellowship (305551/2023‐0). The publication of this article was supported by the Federal University of Pará (UFPA) (PROPESP‐PAPQ 01/2023—QUALIFIED PUBLICATION SUPPORT PROGRAM). There was no external funding to support this research.

## CONFLICT OF INTEREST STATEMENT

The authors declare that they have no known competing financial interests or personal relationships that could have appeared to influence the work reported in this paper.

## Supporting information


Appendix S1–S6.

Figure S1.

Table S1.

Table S2.

Table S3.

Table S4.

Table S5.

## Data Availability

All the requisite information for the construction and compilation of the utilized data is available in the Methods section and Supporting information.
