# Supplementary material for: The future of endemic and threatened birds of the Amazon in the face of global climate change
Source: Ecol Evol. 2024 Mar 18;14(3):e11097. doi: 10.1002/ece3.11097 (PMC10945313; doi:10.1002/ece3.11097)
Supplement: Supplementary file 1 — Appendix S1–S6. [file ECE3-14-e11097-s003.docx]

**SUPPORT INFORMATION**

**THE FUTURE OF ENDEMIC AND ENDANGERED BIRDS OF THE AMAZON IN THE FACE OF GLOBAL CLIMATE CHANGE**

# Appendices

**Appendix S1:** Taxa nomenclature and species occurrence records Cleaning.

The taxa nomenclature follows the Brazilian Committee for Ornithological Records (Pacheco et al, 2021). In addition, in order to use maximum fidelity records to species distribution, we performed data cleaning starting with the removal of geographic coordinates lacking decimals, or with half a decimal degree (0.5); coordinates located outside the continent or in centroids of countries or states; coordinates located in museums or collections, or without species specification. To reduce the uncertainties of geographic bias, one true occurrence record was randomly selected for each resolution grid (pixel).

**References:**

Pacheco J.F., Silveira L.F., Aleixo A., Agne C.E., Bencke G.A., Braco G.A., Q. Piancentini V., et al. Annotated checklist of the birds of Brazil by the Brazilian Ornithological Records Committee—second edition. Ornithology Research. 2021; 29(2): 94–105. Disponível em: doi:10.1007/s43388-021-00058-x .1.

**Appendix S2:** Shared Socioeconomic Pathways (SSPs) scenarios from CMIP6 climate projections.

The Shared Socioeconomic Pathways (SSPs) represent an innovative methodological approach developed to comprehend and explore the intricate interactions between socio-economic factors and global climate change. As a methodological novelty, SSPs scrutinize how diverse socio-economic factors, such as demography, education, and economy, can influence the levels of greenhouse gas emissions (Riahi et al., 2017). The SSP2.4-5, associated with the 'Middle-of-the-Road' trajectory, depicts a path where socio-economic priorities are balanced, but social inequality ranges from moderate to high (O’Neill et al., 2017). This scenario suggests a world with intermediate development, yet with significant disparities among various regions or social groups. In this climate scenario, global CO2 emissions increase until the year 2040, after which emission levels begin to decline. In contrast, the SSP5.8-5, linked to the 'Fossilized Development' narrative, portrays a future where economic values prevail over environmental concerns (O’Neill et al., 2017). In this context, greenhouse gas emissions are high, and they only start declining from the year 2090, resulting in significant intensity in climate change. This scenario reflects a less sustainable trajectory with substantial consequences for the global climate.

In this context, it is crucial to acknowledge that these scenarios do not predict the future but provide valuable insights into possible trajectories that humanity may follow, depending on decisions made over time. These scenarios offer a nuanced understanding of the dynamic interplay between society, economy, and the environment, contributing to the formulation of policies and strategies to address global climate and socio-economic challenges (Riahi et al., 2017).

**References:**

O’Neill, B. C., Kriegler, E., Ebi, K. L., Kemp-Benedict, E., Riahi, K., Rothman, D. S., … Solecki, W. (2017). The roads ahead: Narratives for shared socioeconomic pathways describing world futures in the 21st century. Global Environmental Change, 42, 169–180. doi:10.1016/j.gloenvcha.2015.01.0

Riahi, K., van Vuuren, D. P., Kriegler, E., Edmonds, J., O’Neill, B. C., Fujimori, S., … Tavoni, M. (2017). The Shared Socioeconomic Pathways and their energy, land use, and greenhouse gas emissions implications: An overview. Global Environmental Change, 42, 153–168. doi:10.1016/j.gloenvcha.2016.05.0

**Appendix S3:** Algorithm details and pseudo-absence production

Maximum Entropy (Maxent) (Phillips & Dudik, 2008) produce a presence-background approach and evaluates the relationship between true species occurrence points with study area (background) (Peterson et al, 2011). Random Forest (RDF) (Breiman, 2001), Bayesian Gaussian Process (GAU) (Golding & Purse, 2016), Support Vector Machine (SVM) (Tax & Duin, 2004) and Generalized linear Models (GLM) (Guisan et al, 2002), are presence-absence algorithms that confront true occurrence points (presence points) with points allocated in areas where the species is known not to occur (absence points). In the lacking of known absence points, we used the environmental constraint method, which randomly places samples in areas of low climatic suitability, created by climate models using Bioclim (Engler et al, 2004). In this way, pseudo-absences are generated with a ratio of 1 pseudo-absence point for each presence point. We used masks of the endemic areas where each taxon occurs as allocation limits for the creation of pseudo-absences. This allows for a better calibration of the models, aiming to reduce possible overestimation errors, common in distribution models (Velazco et al, 2020b).

**References:**

Engler R, Guisan A, Rechsteiner L. An improved approach for predicting the distribution of rare and endangered species from occurrence and pseudo-absence data. J Appl Ecol. 2004;41(2):263–74.

Velazco SJE, Ribeiro BR, Laureto LMO, De Marco Júnior P. Overprediction of species distribution models in conservation planning: A still neglected issue with strong effects. Biological Conservation. 2020b; 252: 108822. Disponivel em: doi:10.1016/j.biocon.2020.108822.

**Appendix S4:** Checkerboard and K-fold validation approaches

This type of “block evaluation” structures the training and test data from geographic space, directly evaluating the data and the transferability of the models, thus providing more robust and honest estimates, especially in cases of models with different projections of time and space (Santini et al, 2021). The selection of the optimal cell size of the checkerboard cross-validation was made from tests with different resolutions ranging from twice the resolution of the climate variables to 10 degrees (Velazco et al, 2019), where the criteria of using the resolution is based on: optimizing the parameters of environmental similarity between the training and test data; the spatial autocorrelation between training and test data; and the standard deviation of the number of presence between the training and test data (Velazco et al, 2019). The k-fold method randomly divides the data into blocks (folds) and, at each run, the model is fitted (using one block, k-1 fold) and evaluated from the blocks left out (Fielding & Bell, 1997, Andrade et al, 2020). In this study we chose to use five blocks.

**References:**

Andrade AFA DE, Velazco SJE, De Marco Júnior P. ENMTML: An R package for a straightforward construction of complex ecological niche models. Environmental Modelling & Software. 2020. Disponível em: doi:10.1016/j.envsoft.2019.10461.

Fielding AH, Bell JF. A review of methods for the assessment of prediction errors in conservation presence/absence models. Environmental conservation. 1997; 24(1), 38-49.

Santini, L, Benítez-López, A, Maiorano, L, Čengić, M, Huijbregts, MAJ. Assessing the reliability of species distribution projections in climate change research. *Divers Distrib*. 2021; 27: 1035– 1050. <https://doi.org/10.1111/ddi.13252>.

Velazco SJE, Villalobos F, Galvão F, De Marco Júnior P. A dark scenario for Cerrado plant species: Effects of future climate, land use and protected areas ineffectiveness. Diversity and Distributions. 2019. Disponivel em: doi:10.1111/ddi.12886.

**Appendix S5:** Land Use and Land Cover Models

Both subsets combine the "Top-Down" land demand constraints provided by the official CMIP6 dataset (Stockhause et al, 2021). The use of this official database ensures the use of the information for projections under different SSP-RCP scenarios (Chen et al, 2022). For this study we chose to use the data from the PFT dataset for the present and for the vegetation cover and land use scenarios for the year 2090 corresponding with the addressed optimistic and pessimistic climate scenarios.

After adjusting the spatial resolution and selecting vegetation types that influence the presence of the target species, we overlaid the species distribution models with the Land Use and Land Cover (LULC) models. Subsequently, we identified and selected areas that were common between these models to exclusively illustrate regions climatically suitable for the target taxa, showcasing the projected vegetation associated with these areas.

**References:**

Stockhause, M., Matthews, R., Pirani, A., Treguier, A. M., and Yelekci, O.: CMIP6 data documentation and citation in IPCC's Sixth Assessment Report (AR6), EGU General Assembly 2021, online, 19–30 Apr 2021, EGU21-2886, https://doi.org/10.5194/egusphere-egu21-2886, 2021.

Chen, G., Li, X. & Liu, X. Global land projection based on plant functional types with a 1-km resolution under socio-climatic scenarios. Sci Data 9, 125 (2022).

**Appendix S6:** Protected Areas in Brazil and selection of PAs for the study

In Brazil, there are two main categories of Protected Areas (PAs) according to the Nature Conservation Units System - SNUC (2000): (i) The Strictly Protection Areas (SPA), which has the purpose of maintaining ecosystems free of any human alteration, and can be used for research purposes, and (ii) Sustainable Use Areas (SUA), which can be explored through sustainable management plans. In addition, a large part of the Amazon is made up of Indigenous Territories (IT), where only the native people have the right to live and use the land, according to the Federal Constitution.

In this context, we excluded PAs with size less than 50 km2 to maintain consistency with the spatial scale of SDMs (~5 km) and not to overestimate the number of species in PAs with small sizes (Velazco et al, 2022). In addition, for PAs that encompass marine territories or that extend beyond the study area, we considered only the part included within the Amazon Biome.

**References:**

BRASIL. Ministério do Meio Ambiente. SNUC – Sistema Nacional de Unidades de Conservação da Natureza: Lei nº 9.985, de 18 de julho de 2000; Decreto nº 4.340, de 22 de agosto de 2002; Decreto nº 5.746, de 5 de abril de 2006. Plano Estratégico Nacional de Áreas Protegidas: Decreto nº 5.758, de 13 de abril de 2006. Brasília: MMA, 2011. 76 p.

Velazco SJE, Bedrij NA, Rojas JL, Kelle HA, Ribeiro BR, De Marco P. Quantifying the role of protected areas for safeguarding the uses of biodiversity. Biological Conservation. 2022; 268: 109525.
