# Supplementary material for: The future of endemic and threatened birds of the Amazon in the face of global climate change
Source: Ecol Evol. 2024 Mar 18;14(3):e11097. doi: 10.1002/ece3.11097 (PMC10945313; doi:10.1002/ece3.11097)

Aratinga solstitialis

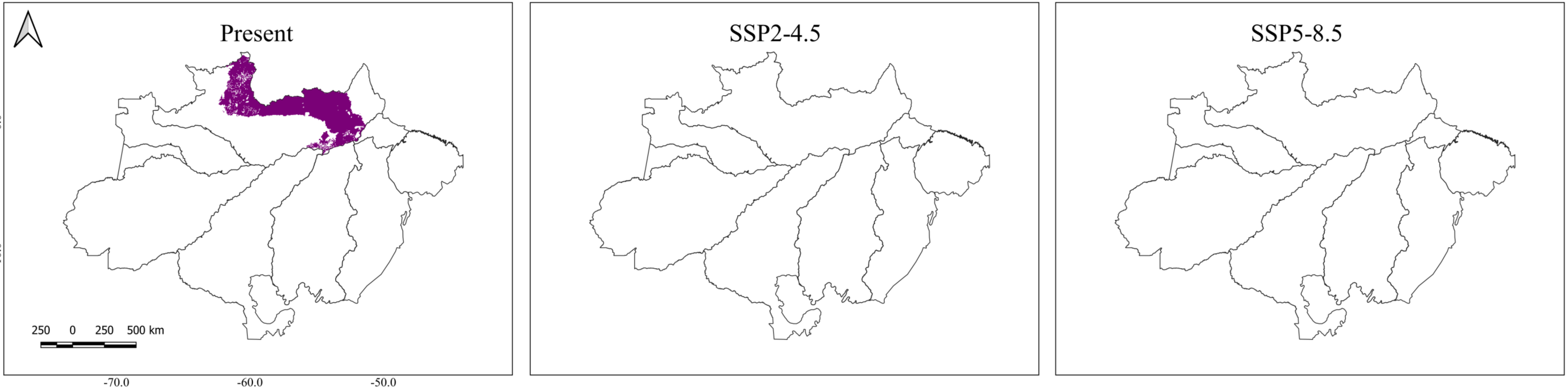

Campylorhamphus multostriatus

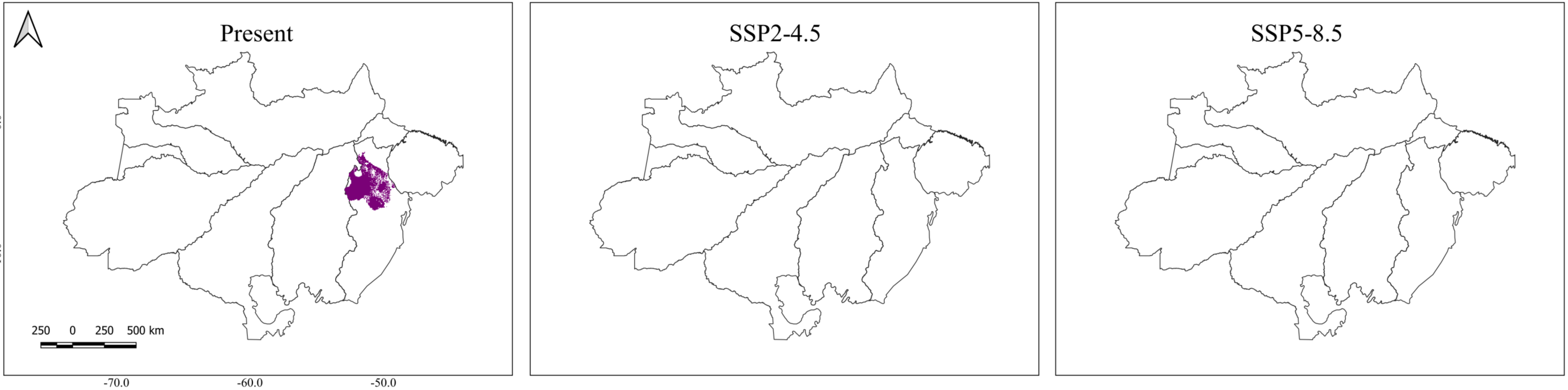

Capito dayi

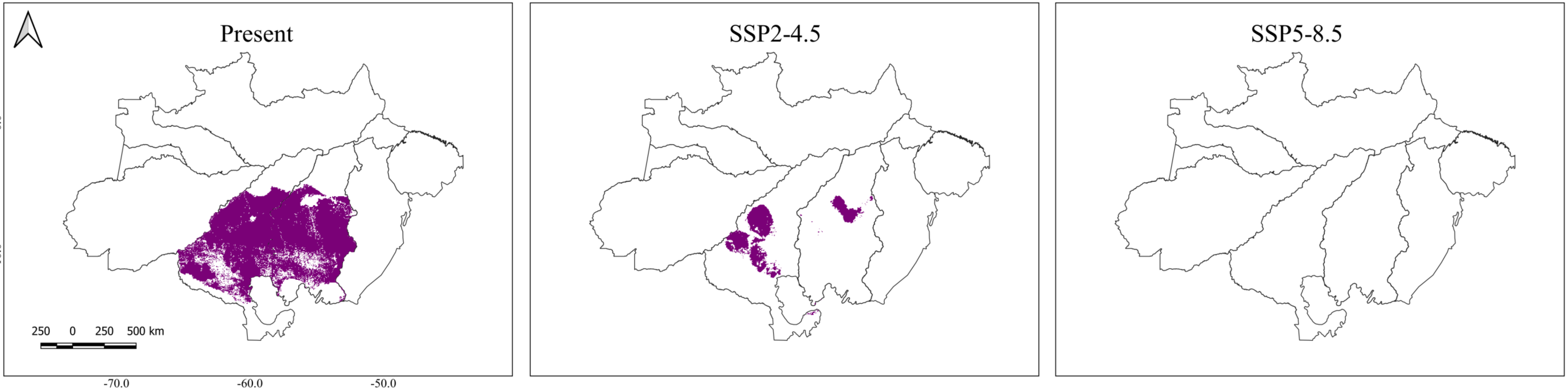

Celeus torquatus pieteroyensi

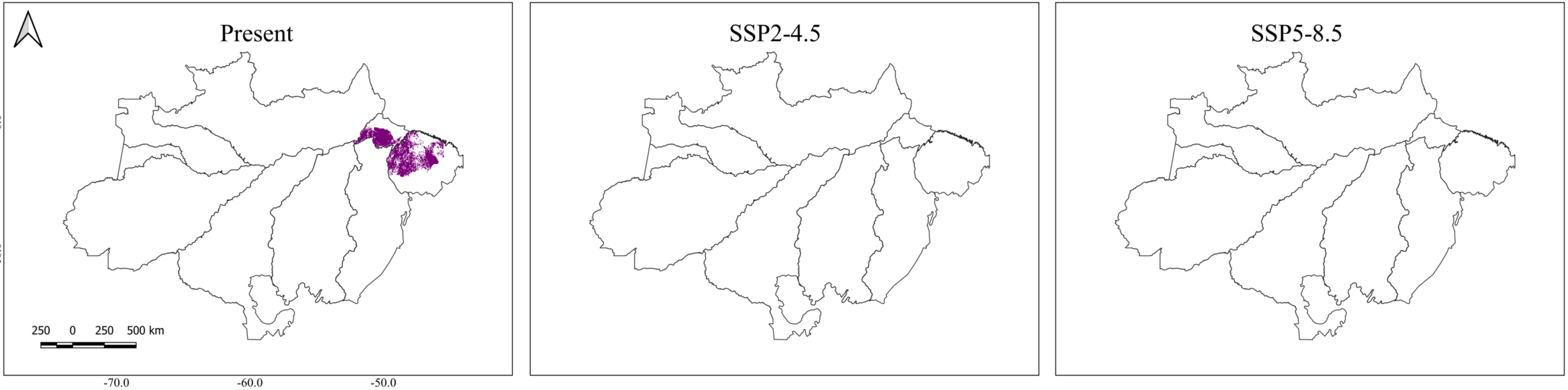

Chamaeza nobilis fulvipectus

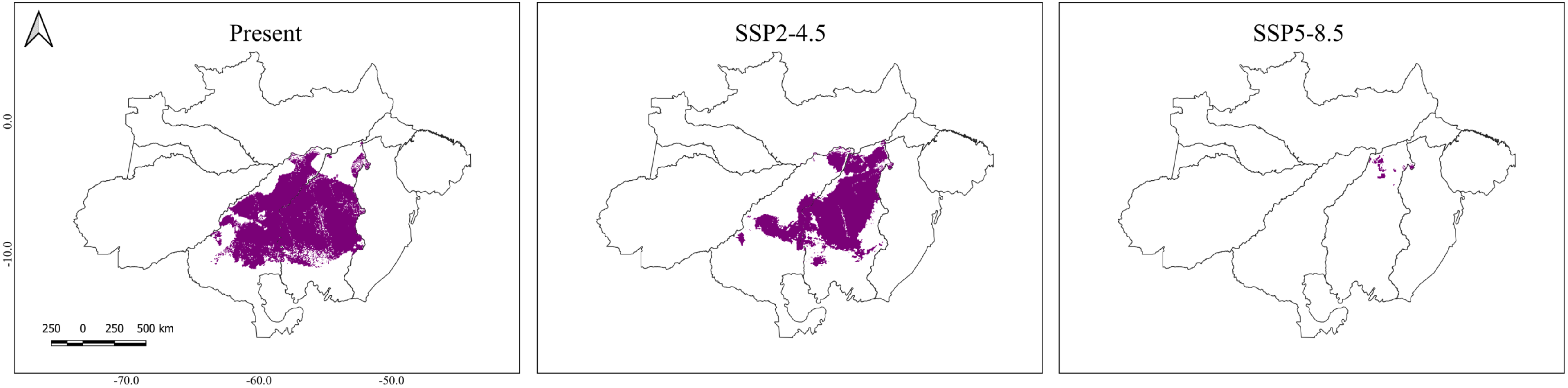

Cranioleuca muelleri

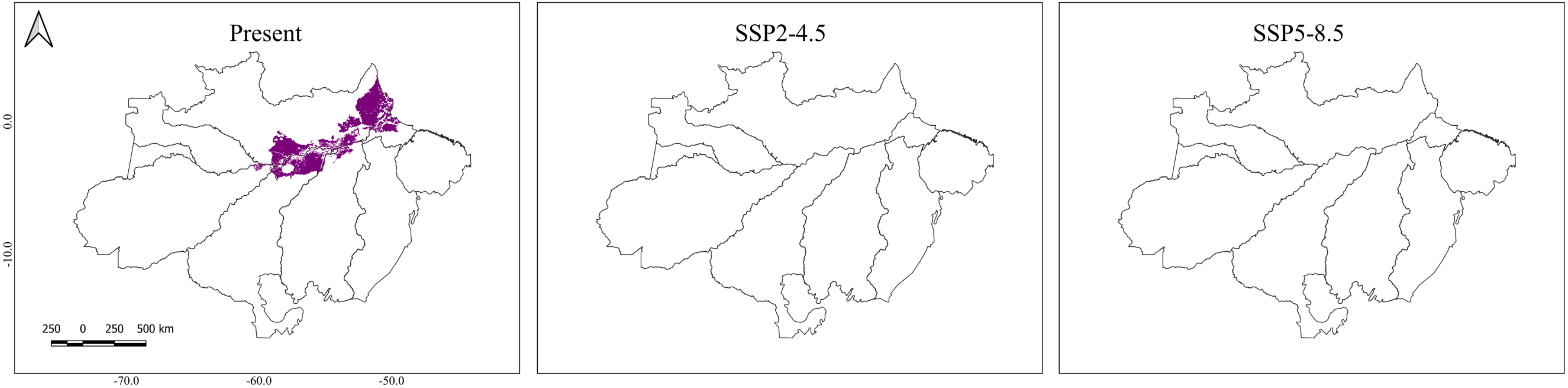

Crax fasciolata pinima

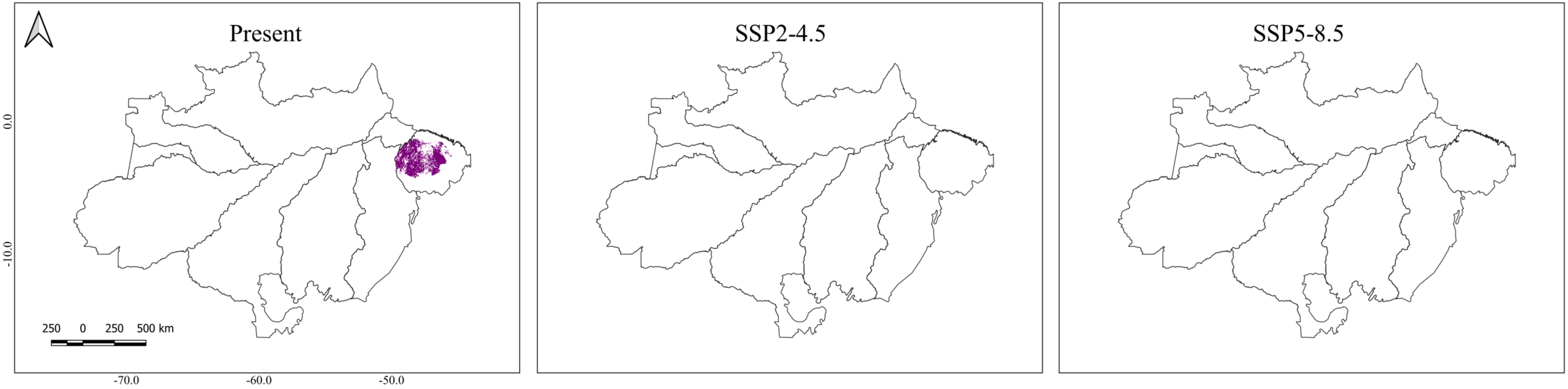

Crax globulosa

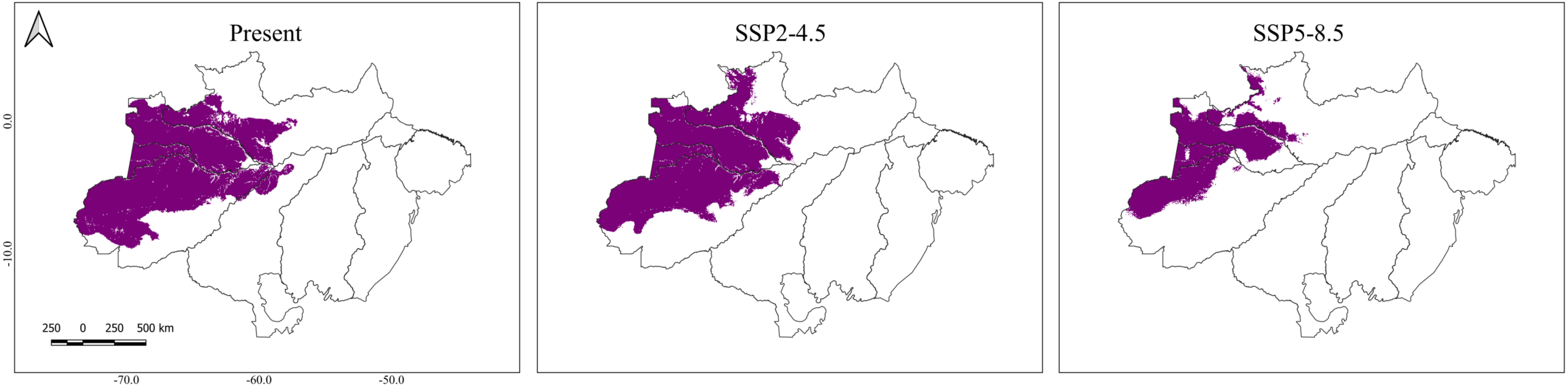

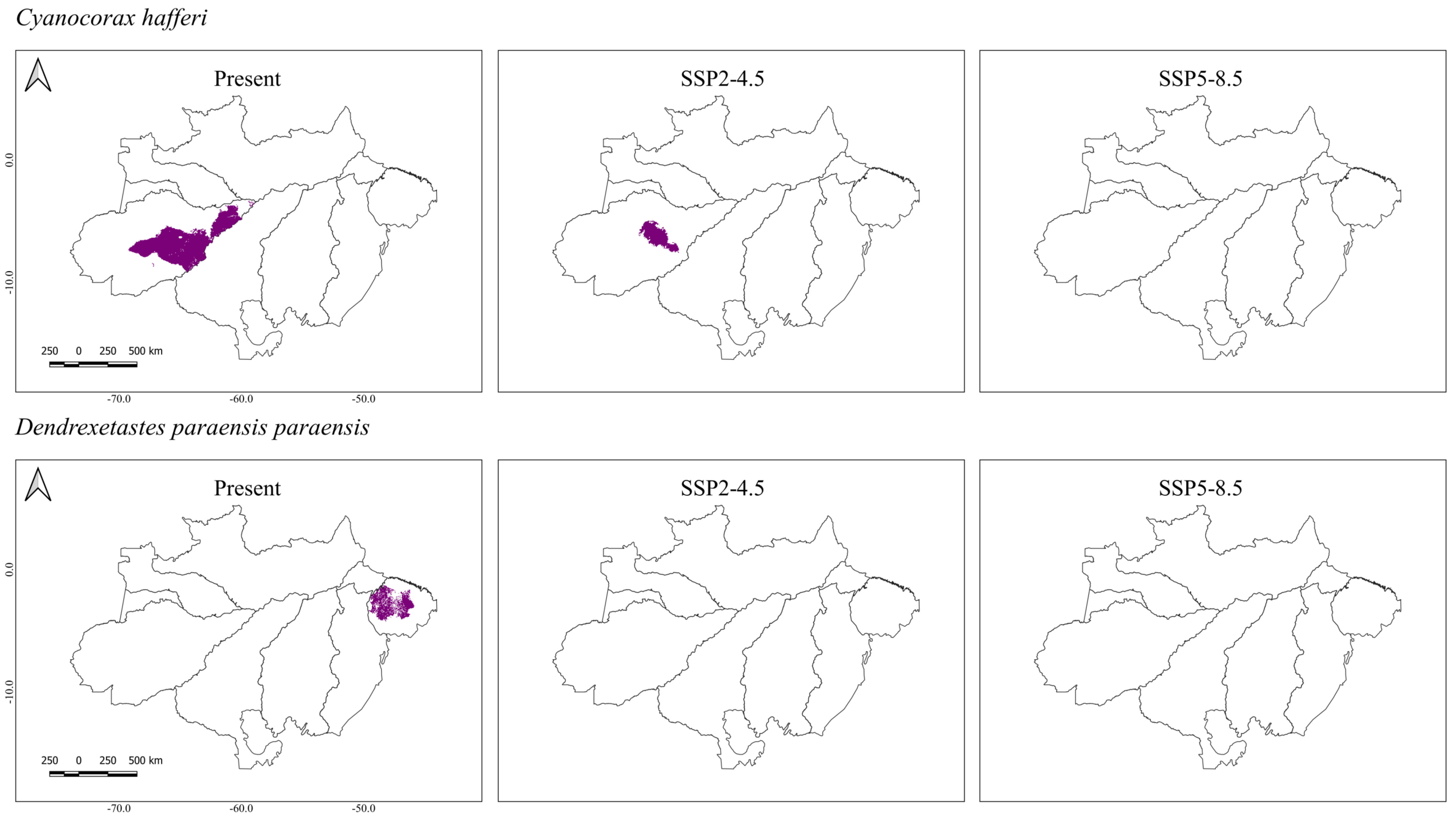
Cyanocorax hafferi
Present
SSP2-4.5
SSP5-8.5
0.0
-10.0
250 0 250 500 km
-70.0
-60.0
-50.0
Dendrexetastes paraensis paraensis
Present
SSP2-4.5
SSP5-8.5
0.0
-10.0
250 0 250 500 km
-70.0
-60.0
-50.0

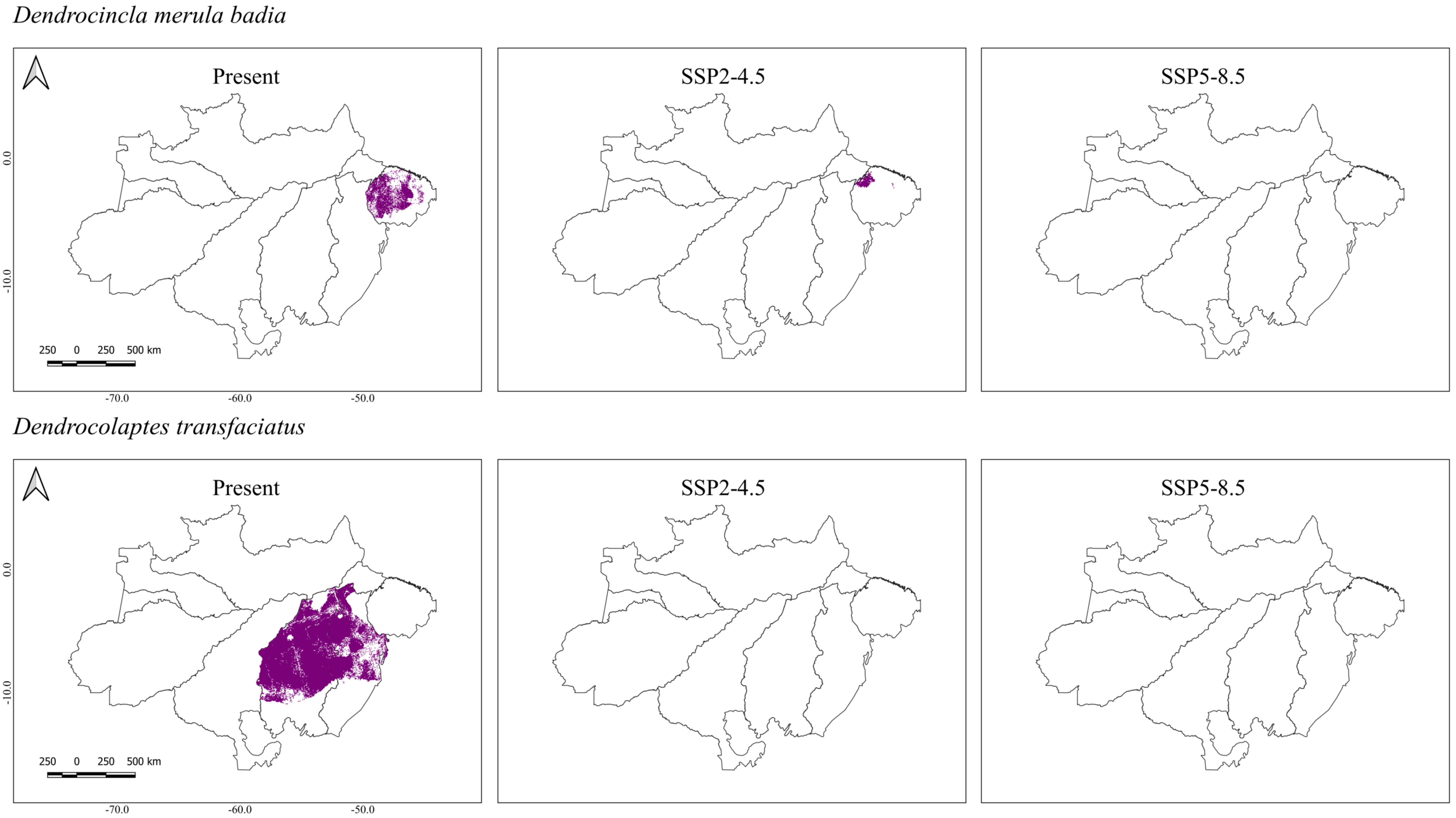
Dendrocincla merula badia
Present
SSP2-4.5
SSP5-8.5
0.0
-10.0
250 0 250 500 km
-70.0
-60.0
-50.0
Dendrocolaptes transfaciatus
Present
SSP2-4.5
SSP5-8.5
0.0
-10.0
250 0 250 500 km
-70.0
-60.0
-50.0

Dendrocolaptes retentus

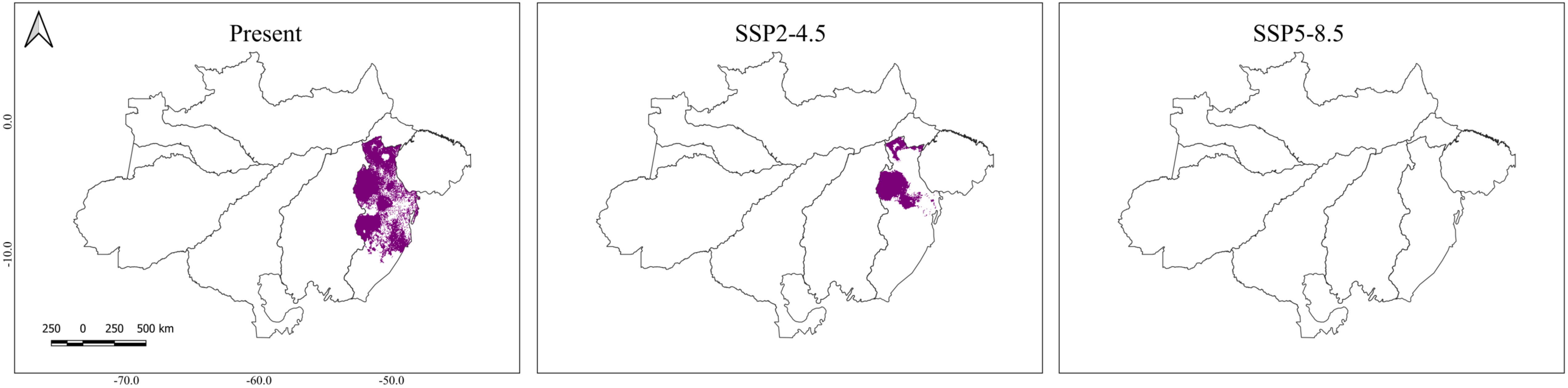

Grallaria varia distincta

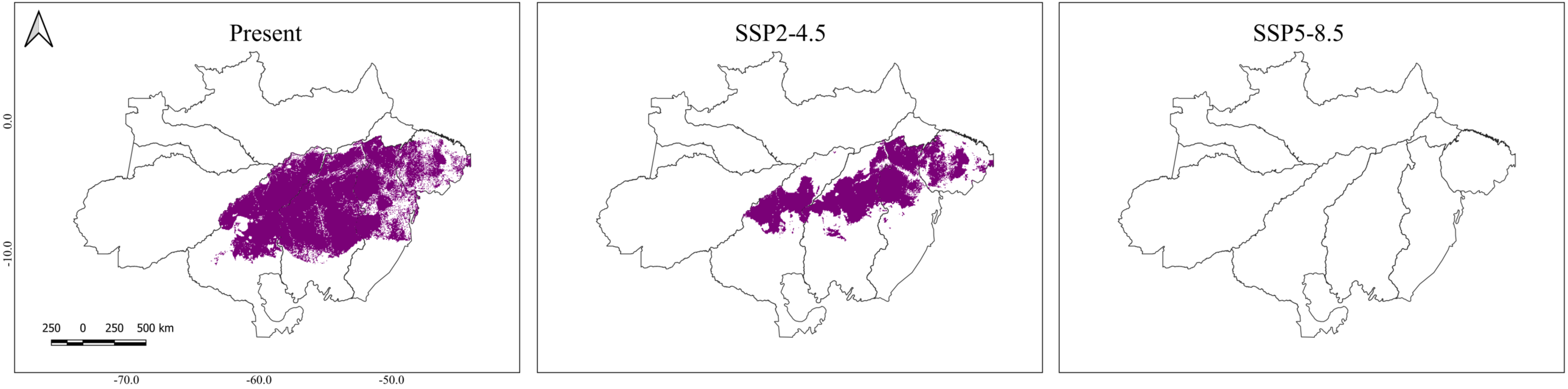

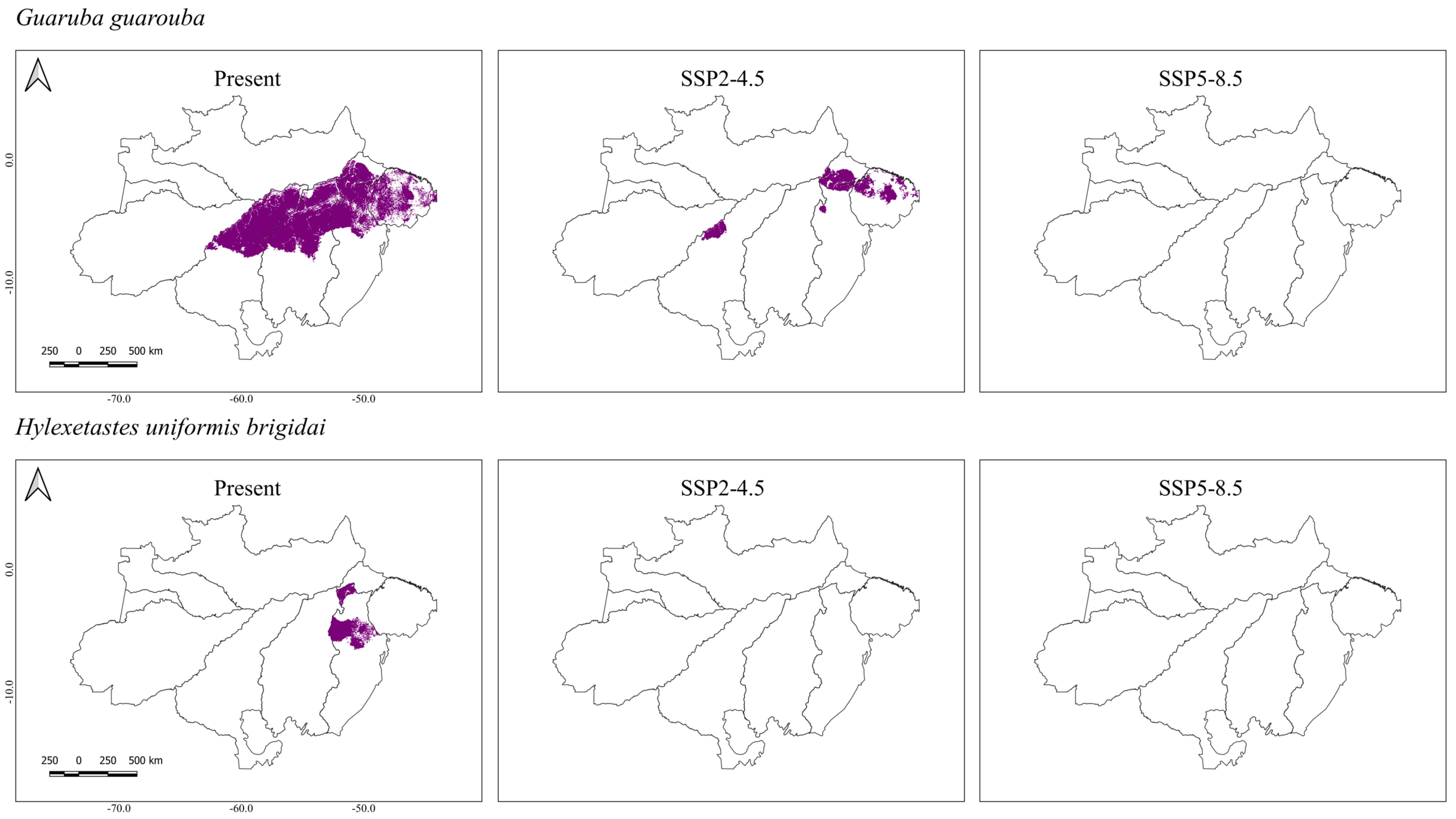
Guaruba guarouba
Present
SSP2-4.5
SSP5-8.5
0.0
-10.0
250
0
250
500 km
-70.0
-60.0
-50.0
Hylexetastes uniformis brigidai
Present
SSP2-4.5
SSP5-8.5
0.0
-10.0
250
0
250
500 km
-70.0
-60.0
-50.0

Hylopezus paraensis

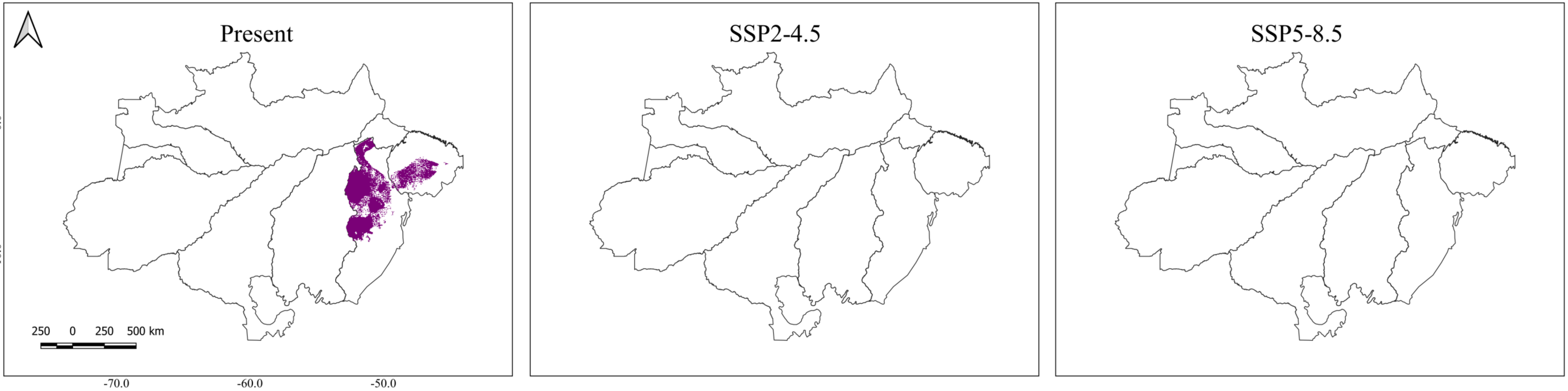

Hypocnemis ochrogyna

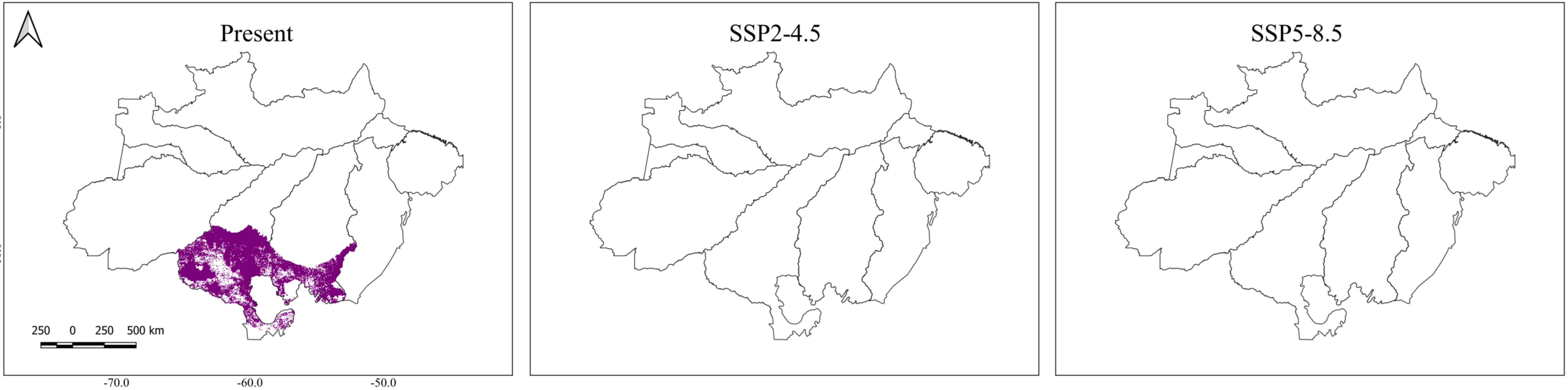

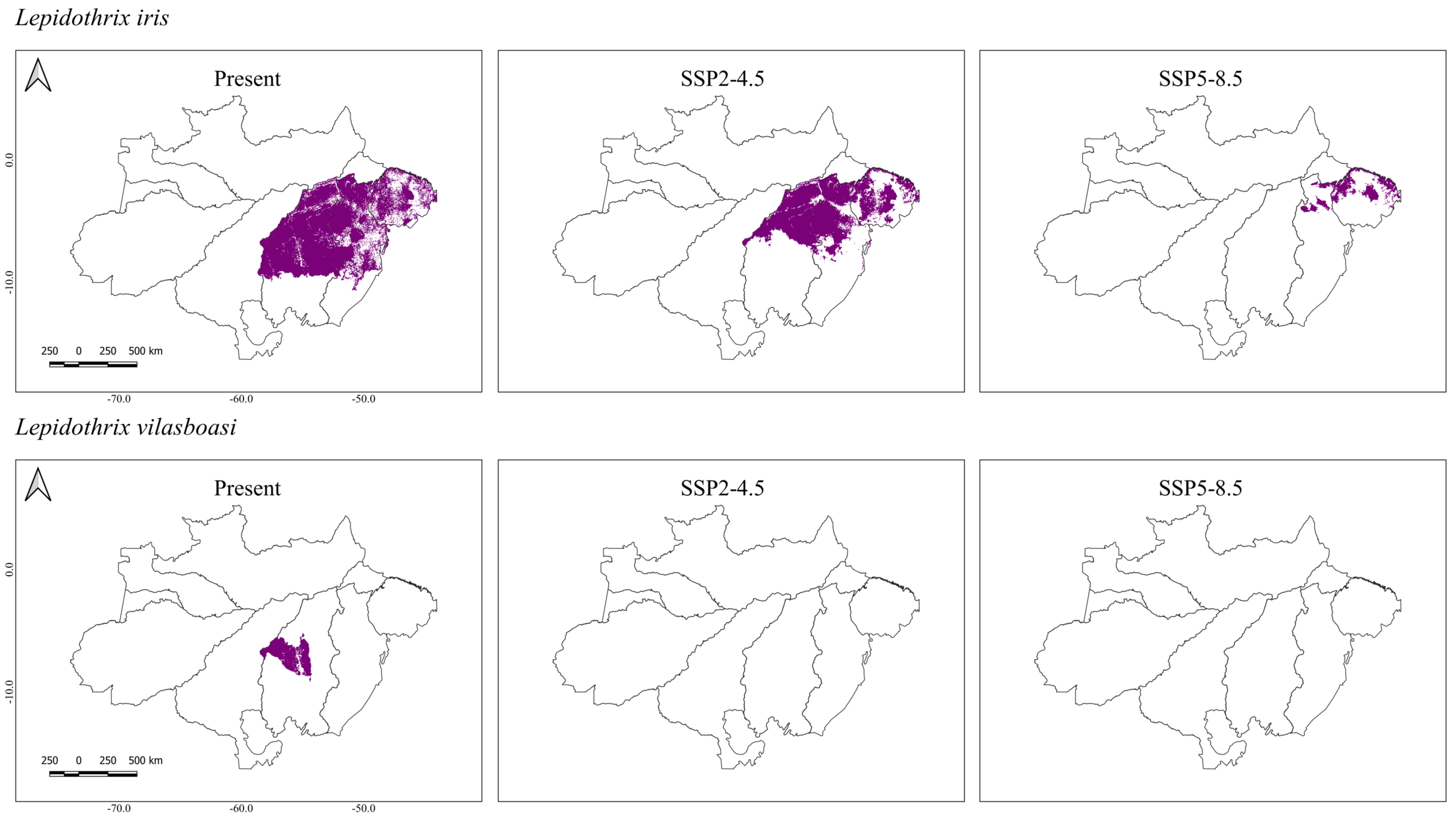

Lepidothrix iris
Present
SSP2-4.5
SSP5-8.5
0.0
-10.0
250 0 250 500 km
-70.0
-60.0
-50.0
Lepidothrix vilasboasi
Present
SSP2-4.5
SSP5-8.5
0.0
-10.0
250 0 250 500 km
-70.0
-60.0
-50.0

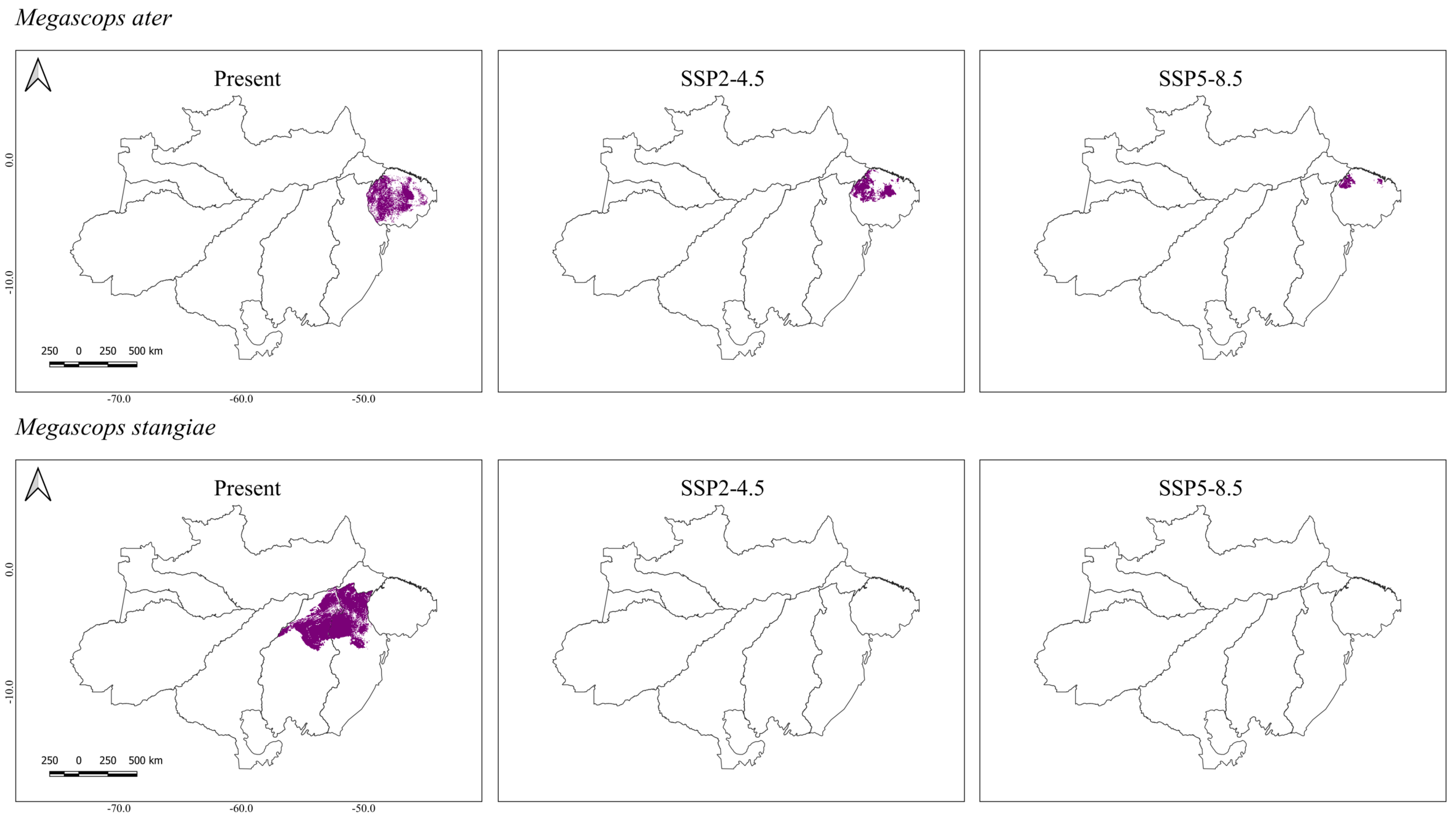

Megascops ater
Present
SSP2-4.5
SSP5-8.5
0.0
-10.0
250 0 250 500 km
-70.0
-60.0
-50.0
Megascops stangiae
Present
SSP2-4.5
SSP5-8.5
0.0
-10.0
250 0 250 500 km
-70.0
-60.0
-50.0

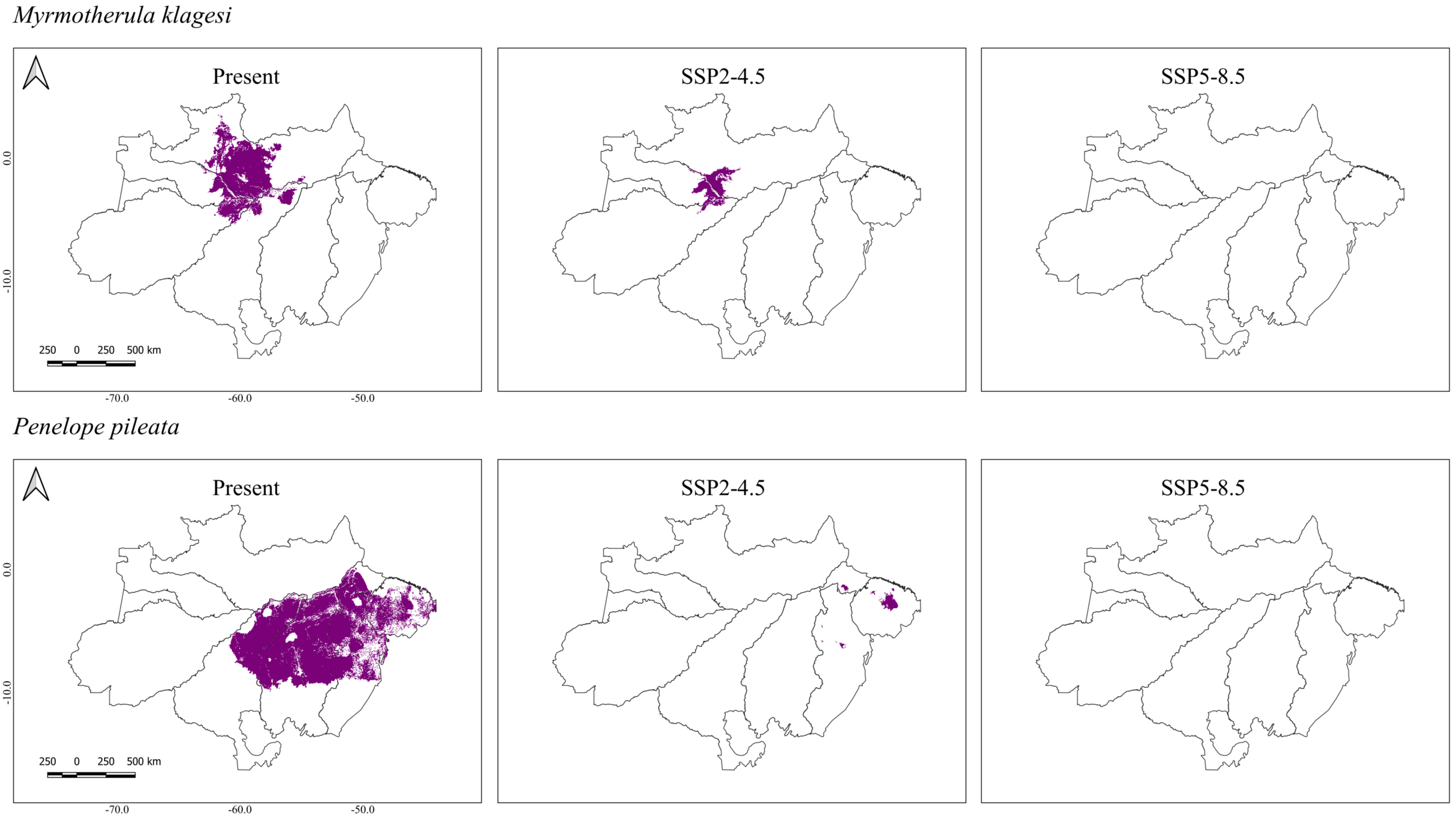
Myrmotherula klagesi
Present
SSP2-4.5
SSP5-8.5
250 0 250 500 km
0.0
-10.0
-70.0
-60.0
-50.0
Penelope pileata
Present
SSP2-4.5
SSP5-8.5
250 0 250 500 km
0.0
-10.0
-70.0
-60.0
-50.0

Phaethornis major

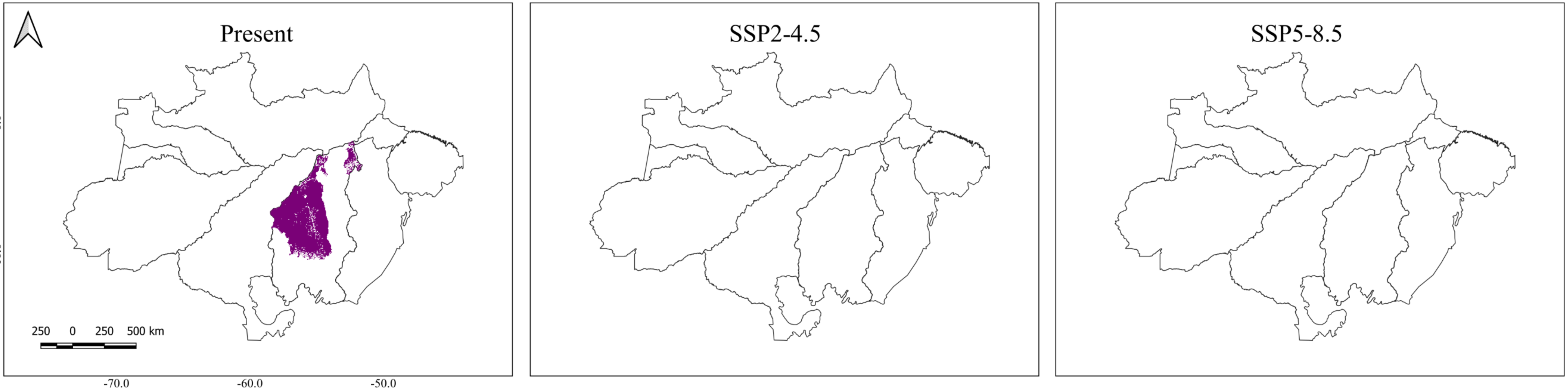

Phlegopsis nigromaculata confinis

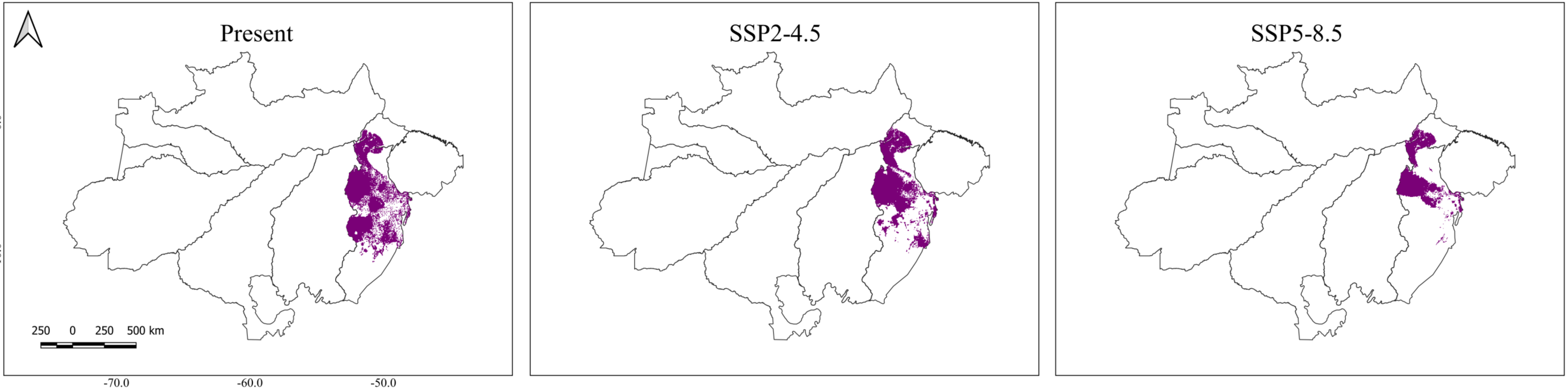

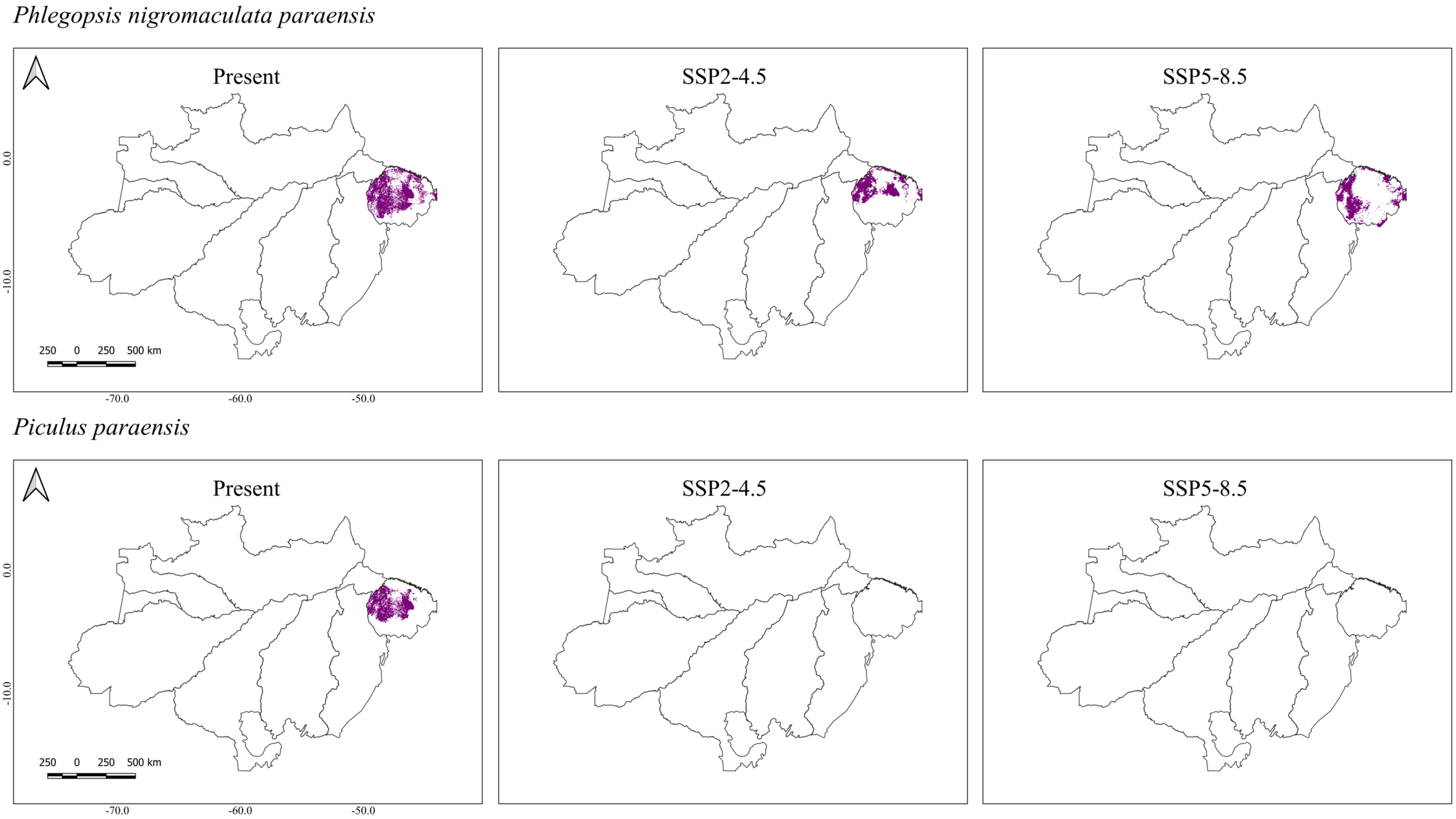
Phlegopsis nigromaculata paraensis
Present
SSP2-4.5
SSP5-8.5
0.0
-10.0
250 0 250 500 km
-70.0
-60.0
-50.0
Piculus paraensis
Present
SSP2-4.5
SSP5-8.5
0.0
-10.0
250 0 250 500 km
-70.0
-60.0
-50.0

Picumnus varzeae

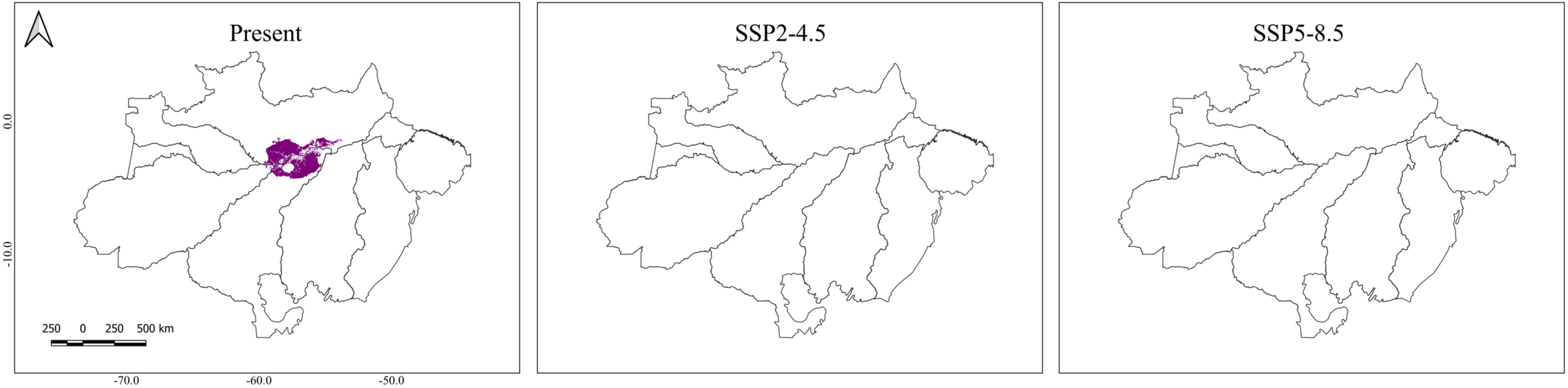

Piprites chloris grisescens

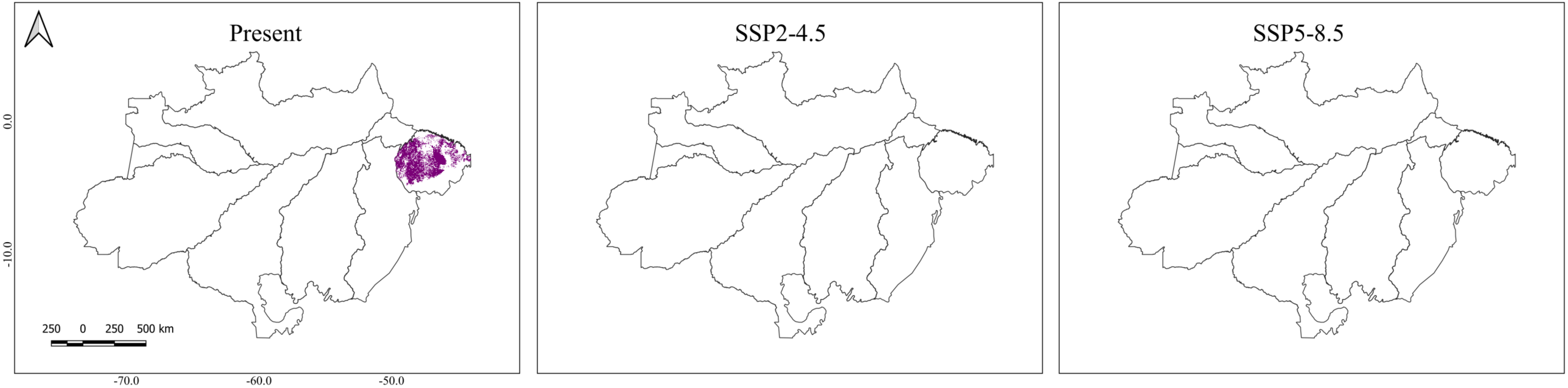

Procnias albus wallacei

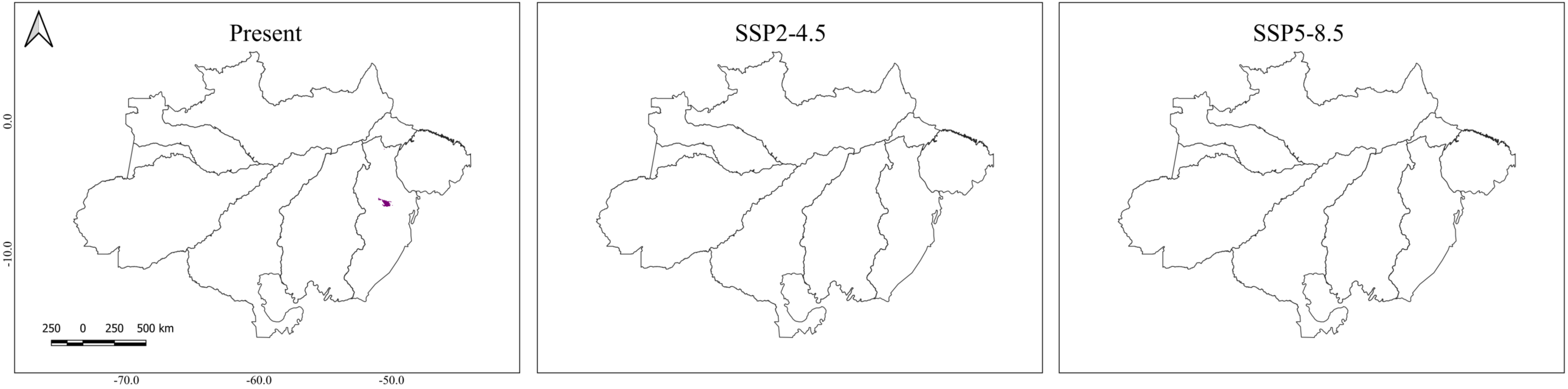

Psophia dextralis

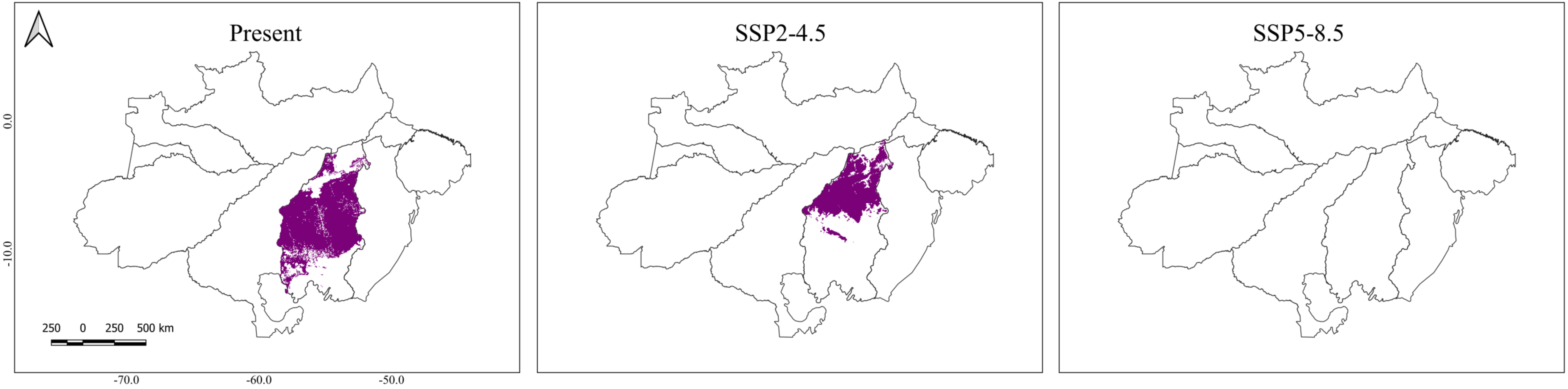

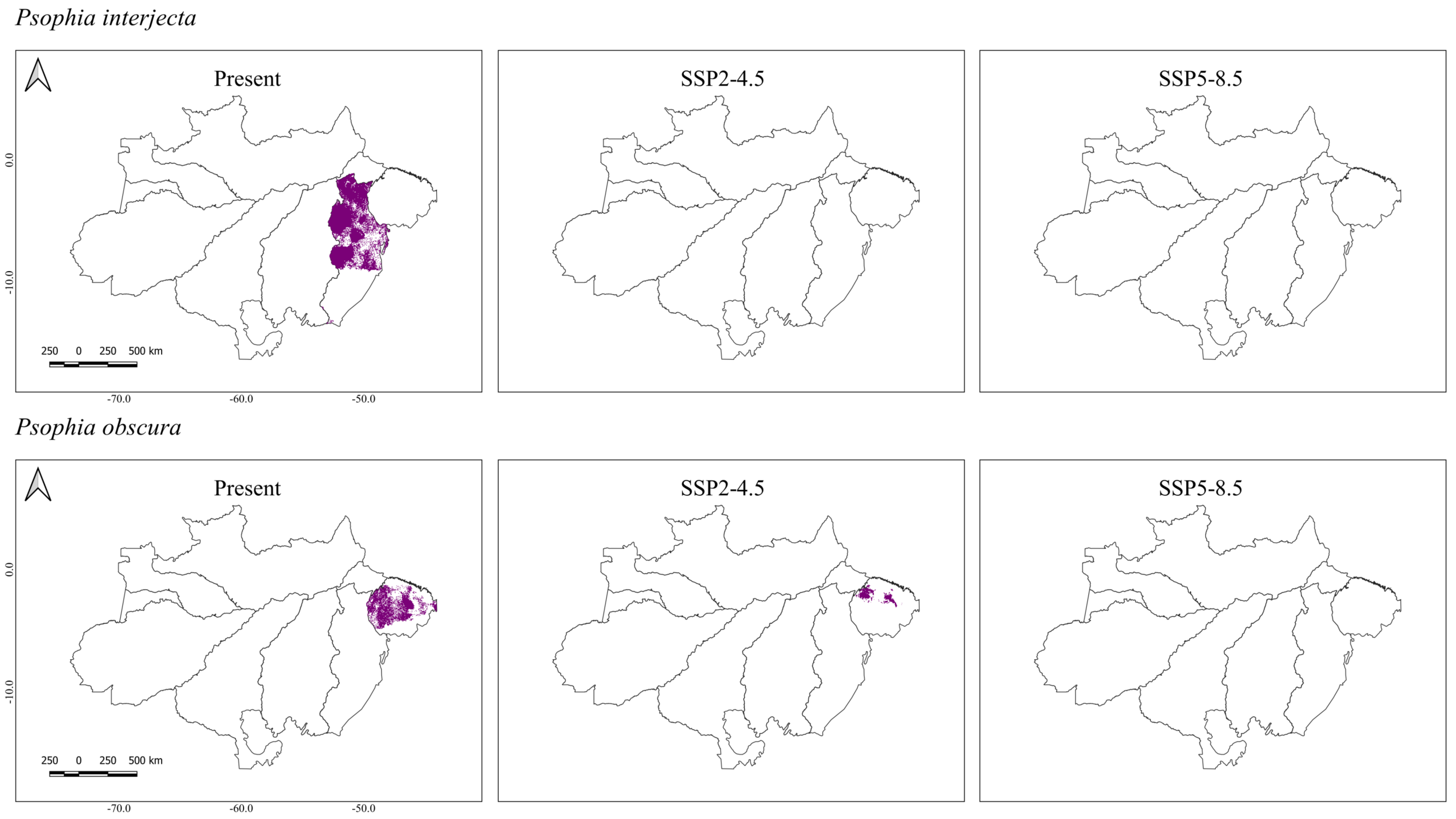
Psophia interjecta
Present
SSP2-4.5
SSP5-8.5
0.0
-10.0
250 0 250 500 km
-70.0
-60.0
-50.0
Psophia obscura
Present
SSP2-4.5
SSP5-8.5
0.0
-10.0
250 0 250 500 km
-70.0
-60.0
-50.0

Pteroglossus bitorquatus bitorquatus

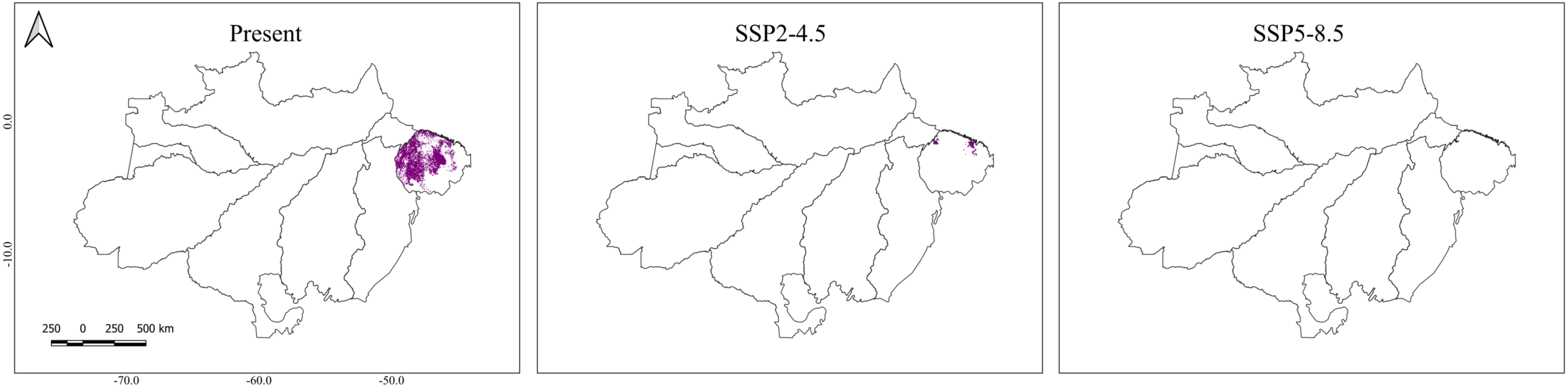

Pyrilia vulturina

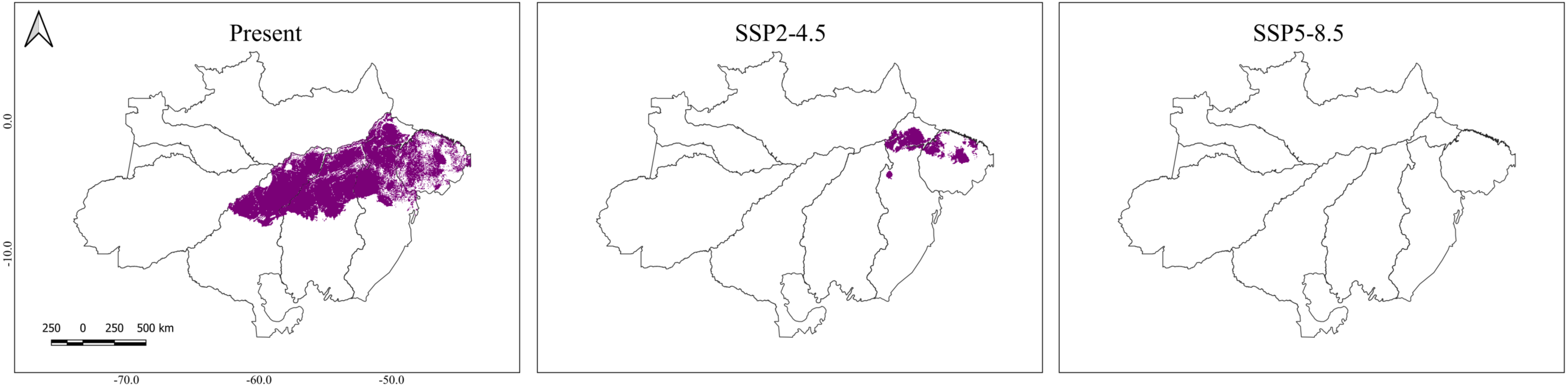

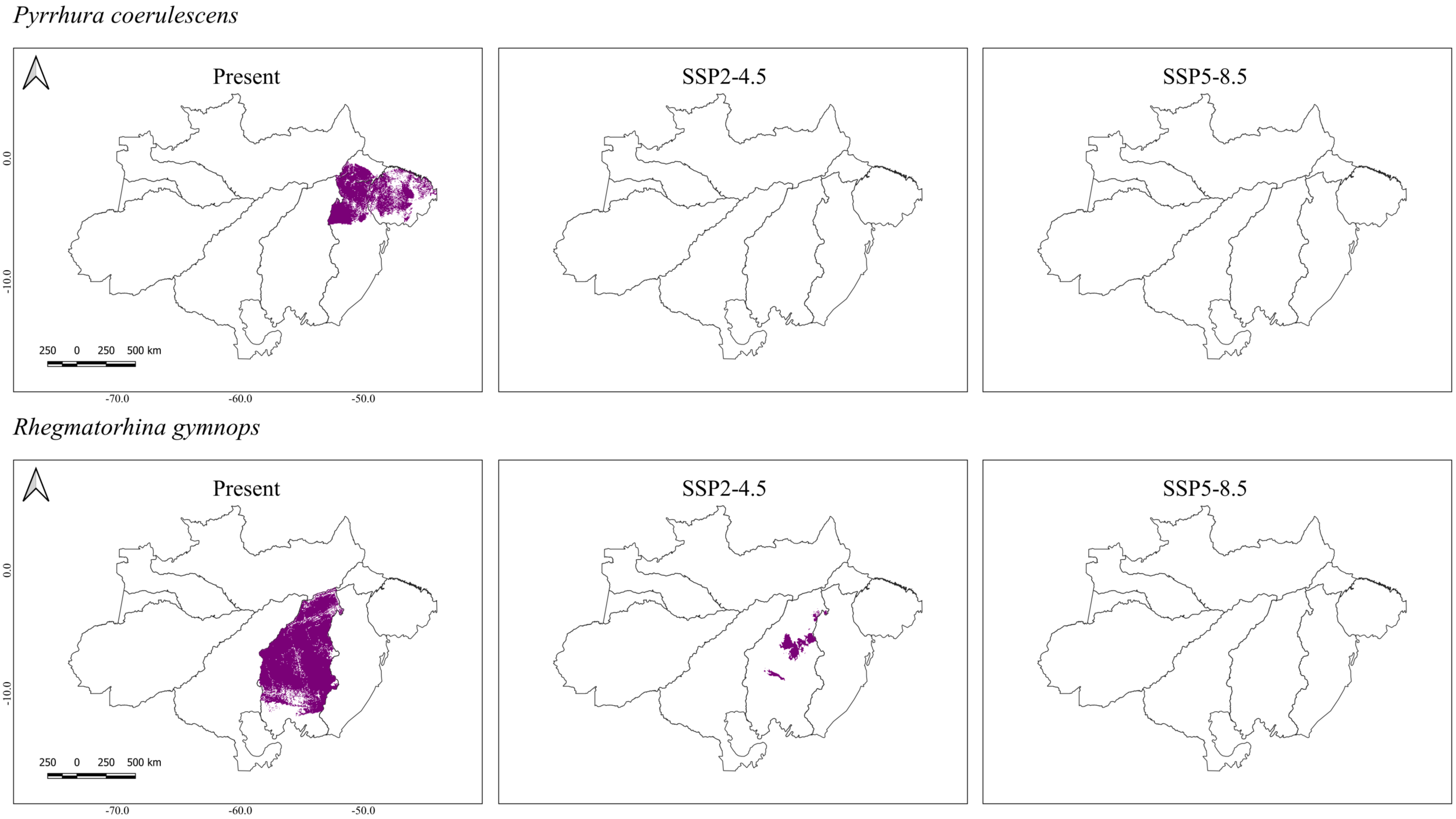

Pyrrhura coerulescens
Present
SSP2-4.5
SSP5-8.5
0.0
-10.0
250 0 250 500 km
-70.0
-60.0
-50.0
Rhegmatorhina gymnops
Present
SSP2-4.5
SSP5-8.5
0.0
-10.0
250 0 250 500 km
-70.0
-60.0
-50.0

Synallaxis kollari

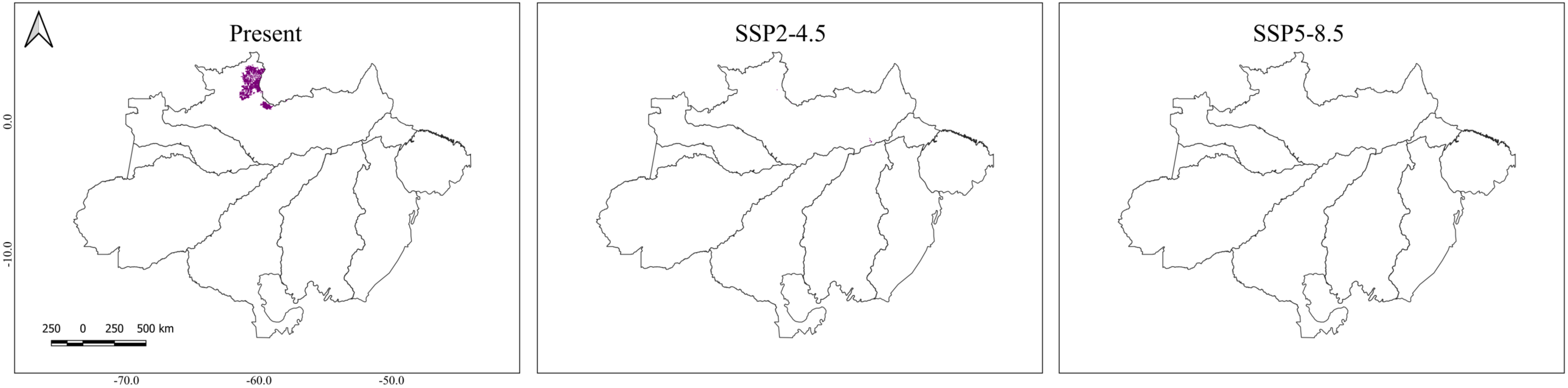

Tangara velia signata

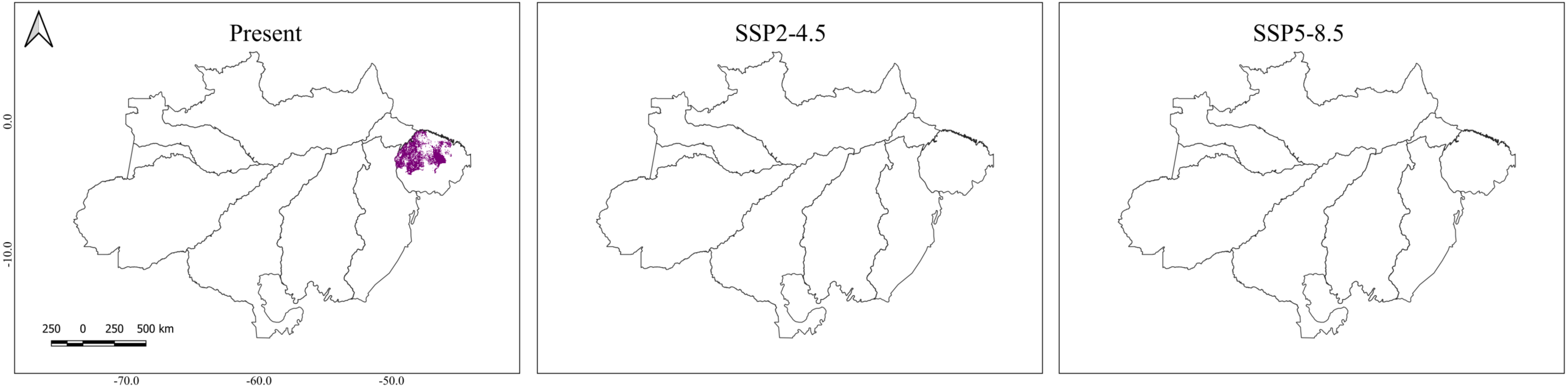

Tinamus tao tao

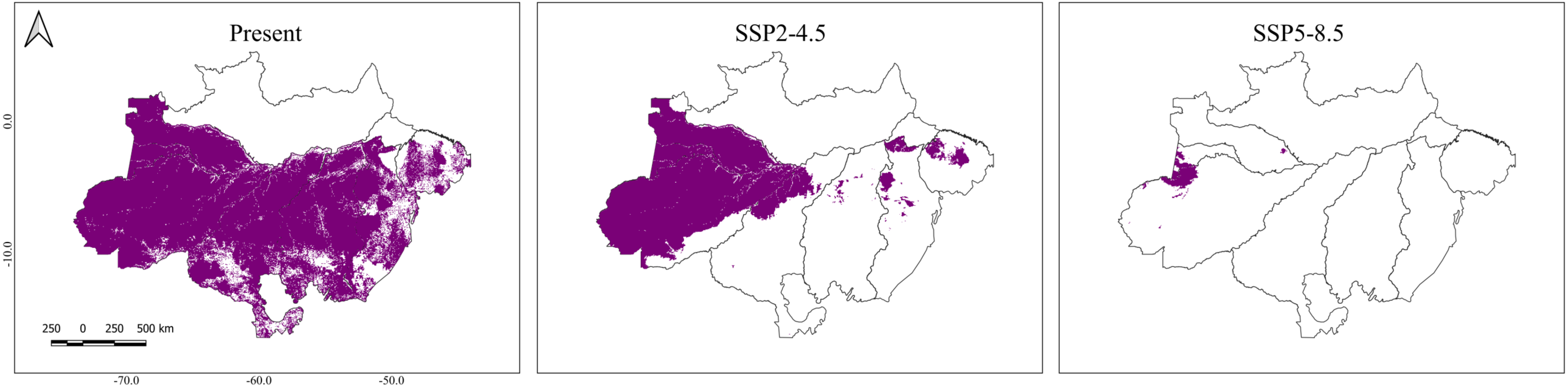

Tunchiornis ochraceiceps rubrifrons

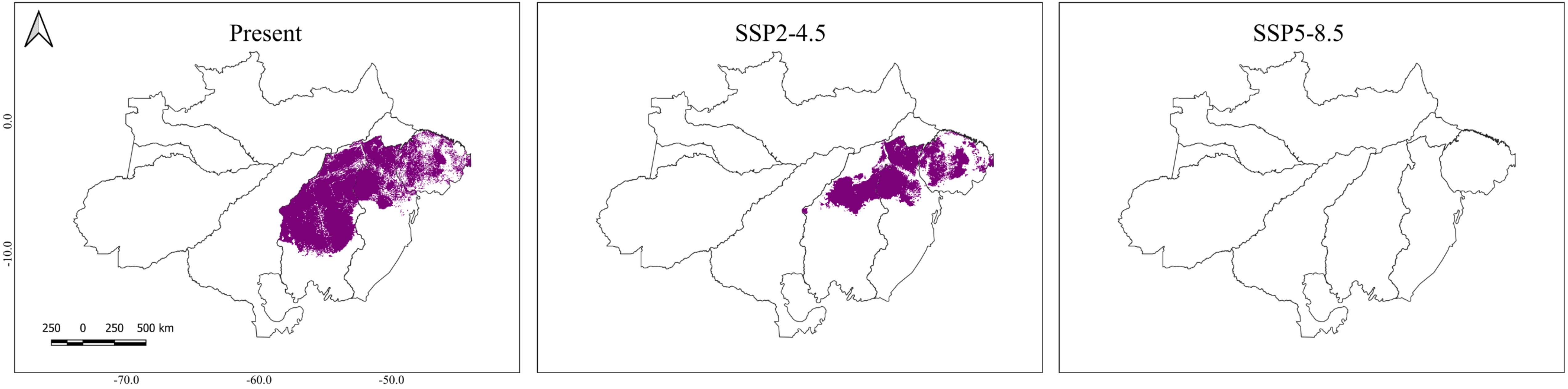

Xiphocolaptes carajaensis

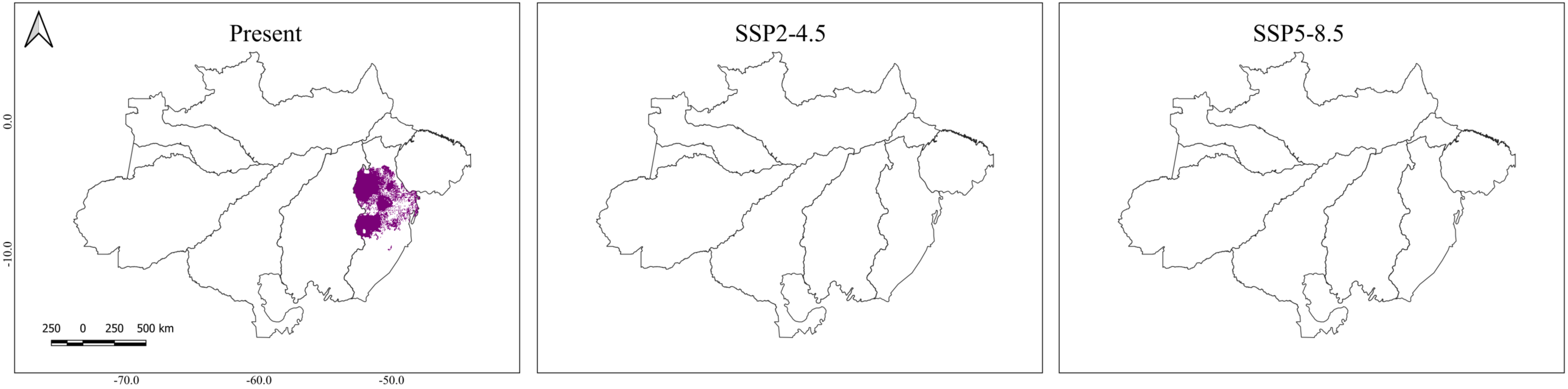

Supplement: Supplementary file 2 — Figure S1. [file ECE3-14-e11097-s004.pdf]
